# *Artemisia annua* L. polysaccharide improves the growth performance and intestinal barrier function of broilers challenged with *Escherichia coli*

**DOI:** 10.3389/fmicb.2024.1390815

**Published:** 2024-04-30

**Authors:** Shiwei Guo, Binlin Shi, Yuanyuan Xing, Yuanqing Xu, Xiao Jin, Lei Hong, Shengnan Zhang, Min Qiao, Sumei Yan

**Affiliations:** College of Animal Science, Inner Mongolia Agricultural University, Hohhot, China

**Keywords:** *Artemisia annua L. polysaccharide*, broiler, *Escherichia coli*, intestinal barrier function, microbiota

## Abstract

With the high intensification of poultry breeding, a series of diseases caused by pathogenic bacteria threaten the health of poultry and human. Among them, poultry diseases induced by *Escherichia coli* cause significant economic loss every year. The aim of this study was to investigate the effects of dietary supplementation with *Artemisia annua* L. polysaccharide (AAP) on the growth performance and intestinal barrier function of broilers with *Escherichia coli* (*E. coli*) challenge. A total of 256 one-day-old chicks were randomly assigned to four treatment groups: control group (fed basal diet), AAP group (fed basal diet supplemented with AAP), *E. coli* group (fed basal diet and orally administered *E. coli*), AAP + *E. coli* group (fed basal diet supplemented with AAP and orally administered *E. coli*). Dietary AAP supplementation elevated the BW, ADG and ADFI in non-challenged broilers. AAP also increased the apparent metabolic rate of EE and Ca in *E. coli*-challenged broilers. Moreover, AAP not only enhanced the serum IgA content but also decreased the serum and jejunum content of IL-6, as well as the jejunum level of IL-1β in non-challenged broilers. AAP also down-regulates the mRNA level of inflammatory factors (*IL-1β*, *IL-6*, and *TNF-α*) by inhibiting the mRNA expression of *TLR4* and *MyD88* in intestinal NF-κB signaling pathway of *E. coli*-challenged broilers. Meanwhile, AAP up-regulates the activity and mRNA level CAT by down-regulating the mRNA level of *Keap1* in intestinal Nrf2 signaling pathway of *E. coli*-challenged broilers, and decreased serum MDA concentration. AAP significantly elevated the mRNA level of *CAT*, *SOD* and *Nrf2* in jejunal of non-challenged broilers. Interestingly, AAP can improve intestinal physical barrier by down-regulating serum ET content, increasing the jejunal villus height/crypt depth (VH/CD) and *ZO-1* mRNA level in broilers challenged by *E. coli*. AAP also elevated the VH/CD and the mRNA level of *Occludin*, *ZO-1*, *Mucin-2* in non-challenged broilers. Importantly, AAP reshaped the balance of jejunum microbiota in *E. coli*-challenged broilers by altering α diversity and community composition. In summary, AAP ameliorated the loss of growth performance in broilers challenged with *E. coli*, probably by regulating the intestinal permeability and mucosa morphology, immune function, antioxidant ability, and microbiota.

## 1 Introduction

The health of the gastrointestinal tract is crucial for the overall health and production performance of poultry. Firstly, it is a part of the digestive system responsible for breaking down food, absorbing nutrients, and excreting undigested food. The intestinal tract is composed of the mucosal layer and epithelial cells, which effectively block the invasion of pathogenic bacteria through tight junctions and adherent junctions between the cells. In addition, the presence of gut-associated lymphoid tissue (GALT) and immune cells further enhances the defensive ability of the intestinal tract. GALT is the main part of intestinal lymphoid tissue and can produce antibodies and cytokines to mount immune responses against invading pathogens. Immune cells, including lymphocytes, macrophages, and other white blood cells, play a crucial role in the intestinal tract. These cells possess the ability to recognize and attack invading pathogens, thus protecting poultry from infection (Berkes et al., 2003; Yegani and Korver, 2008).

When the gastrointestinal tract is infected with pathogenic bacteria such as *Escherichia coli* (*E. coli*), the intestinal barrier may be disrupted, leading to the invasion of the bacteria into the body and triggering an inflammatory response and infection. The negative effects of *E. coli* on poultry are primarily manifested in intestinal inflammation, disrupted digestive function, decreased growth performance, and increased mortality. These consequences not only impact the health and production performance of the animals but also result in substantial economic loss for the poultry farming industry (Croxen et al., 2013; Gomes et al., 2016). In recent years, research on mitigating the negative impacts of avian *E. coli* has garnered increasing attention. It has been reported that dietary live yeast and mannan-oligosaccharide supplementation can effectively attenuate *E. coli*-induced intestinal disruption in broilers by reducing intestinal inflammation and barrier dysfunction (Wang et al., 2016). Furthermore, Kumari et al. (2023) discovered that added drinking water with *Aloe vera* leaf extract could mitigate the detrimental effects of *E. coli*-challenge on broilers by enhancing antioxidant level and bolstering cellular immune response. In addition, studies showed that dietary hydrolyzed wheat gluten supplementation ameliorated intestinal barrier dysfunction of broilers challenged with *E. coli* O78 (Wu et al., 2022b).

In recent years, plant-derived polysaccharides have been widely studied in poultry production, which can relieve various stress and improve intestinal barrier function (Guo et al., 2022b). It has been reported that dietary Gan Cao (*Glycyrrhiza uralensis Fisch*) polysaccharide improves growth performance, immune function, gut microflora and intestinal health of broiler chickens (Wu et al., 2022c; Zhang et al., 2022c). Qiao et al. (2022b) also found that polysaccharides derived from *Astragalus membranaceus* and *Glycyrrhiza uralensis* improved the growth performance of broilers by enhancing intestinal health and modulating gut microbiota. Moreover, dietary *Astragalus* polysaccharide supplementation has been found to alleviate necrotic enteritis in broiler chickens by balancing Th17/Treg response and regulating gut microbiota composition (Song et al., 2022). Besides, dietary *Caulis Spatholobi* polysaccharide alleviated the immunosuppression induced by cyclophosphamide in broilers though regulating immunity, intestinal mucosal barrier function, and intestinal microbiota (Cui et al., 2022). Previous studies demonstrated that dietary supplementation *Artemisia* polysaccharides have beneficial effects on broilers. It was found that dietary supplementation with *Artemisia argyi* polysaccharide improved immune and antioxidative functions in broilers (Zhang et al., 2022b). It has been reported that *Artemisia ordosica* polysaccharide ameliorated LPS-induced growth inhibition and intestinal injury in broilers through enhancing immune regulation and antioxidant capacity (Xing et al., 2023).

*Artemisia annua* L. (*A. annua*) is a species of *Artemisia* within the *Asteraceae* family, is widely distributed (Ding et al., 2020) and is renowned for its rich variety of active substances, including sesquiterpenoids, flavonoids, coumarins, and volatile oils (Das et al., 2020; Fu et al., 2020), consequently, it possesses numerous biological functions, including anti-inflammatory, antioxidant, antibacterial, antiviral, antitumor anti-malarial and immune regulatory activity (Graham et al., 2019; Stan, 2020; Han et al., 2022; Chebbac et al., 2023; Wu et al., 2023). In poultry, previous studies have shown that *A. annua* aqueous extract can promote intestine immunity and antioxidant function (Guo et al., 2022a), inhibit the reproduction of *E. coli* and promote the proliferation of *Lactobacillusla* in broilers (Guo et al., 2023). In addition, adding *A. annua* and its extracts into diets can effectively relieve heat stress, immune stress and oxidative stress in broilers (Song et al., 2017, 2018; Wan et al., 2017, 2018; Choi et al., 2020). Addition of *A. annua* extract to Nile tilapia diets modulated the intestinal microbiota and altered the abundance of specific bacteria (*Fusobacteriaceae*, *Stenotrophomonas*, and *Clostridium*), thereby improving growth performance (Soares et al., 2022). Notably, the polysaccharide, being a bioactive substance in *A. annua*, has been found to possess antitumor activity (Yan et al., 2019), along with antioxidant and immunomodulatory properties, as confirmed by *in vitro* experiments (Zhang et al., 2022a). The stable structures and properties of polysaccharides, as macromolecular compounds, contribute to their notably high safety profile. With a mature extraction and purification technology specific to *Artemisia* (Xing et al., 2020; Zhang et al., 2022b), polysaccharides are poised for large-scale production and application. However, it has not been reported whether *A. annua* polysaccharide is beneficial to broilers so far. In this study, oral administration of *E. coli* was used to establish a challenge model, in order to investigate the effect of dietary supplementation of *A. annua* polysaccharide on growth performance and intestinal barrier function of broilers challenged with *E. coli*, and provide theoretical basis for the application of *A. annua* polysaccharide in poultry production.

## 2 Materials and methods

### 2.1 Animal ethics statement

All animal experiments were approved by the Inner Mongolia Agricultural University Animal Care and Use Committee, Hohhot, P. R. China (approval number: NND2021090).

### 2.2 Preparation of *Artemisia annua* L. polysaccharide

*Artemisia annua* L. (*A. annua*) plants were collected from Hohhot, Inner Mongolia, China. The collected plants were washed with distilled water and air-dried in the shade at room temperature. A quantity of 500 g of *A. annua* powder was taken and subjected to ultrasound treatment using petroleum ether for 30 min to remove fat. The resulting powder was then passed through a 60-mesh sieve. After the natural volatilization of petroleum ether, *A. annua* polysaccharide extraction was performed using a complex enzyme with a solid–liquid ratio of 1:30. The enzyme mixture consisted of cellulase, pectinase, and papain, added at proportions of 23.2, 19.7, and 15.6%, respectively. The mixture was incubated in a constant temperature shaker at 50°C and 150 rpm for 3 h. Subsequently, it was transferred to a 70°C water bath for 1 h to inactivate the enzymes. The resulting mixture was filtered and concentrated, followed by the addition of anhydrous ethanol into the concentrate at a 4:1 volume ratio. The solution was refrigerated in a 4°C refrigerator for 48 h, then the solution was centrifuged at 3000 × g for 5 min, and the precipitate was collected. The collected precipitate was washed with anhydrous ethanol and acetone, each three times. Subsequent to the washing, the precipitate was freeze-dried to obtain the *A. annua* polysaccharide. The total soluble sugar content of AAP was measured at 397.42 mg/g, while its molecular weight was identified as 14.639 kDa. AAP was consisted of rhamnose, arabinose, galactose, glucose, mannose, galacturonic acid, and glucuronic acid, with a molar ratio of 2.93:3.75:7.44:58.47:20.87:5.49:1.07. According to the previous study, the appropriate dosage of AAP is 750 mg/kg (Zhang, 2023).

### 2.3 Bacterial strains and culture

*Escherichia. coli* O78 (CVCC1490) was obtained from CVCC (China Veterinary Culture Collection Center). The strain was cultured at 37°C in Luria-Bertani (LB) broth (Guangdong Huankai Microbial Sci. & Tech. Co., Ltd., Guangzhou, China) for 24 h to reach a final concentration of 1.0 × 10^12^ CFU/mL in sterile LB liquid medium.

### 2.4 Experimental design and diets

**Figure 1:**
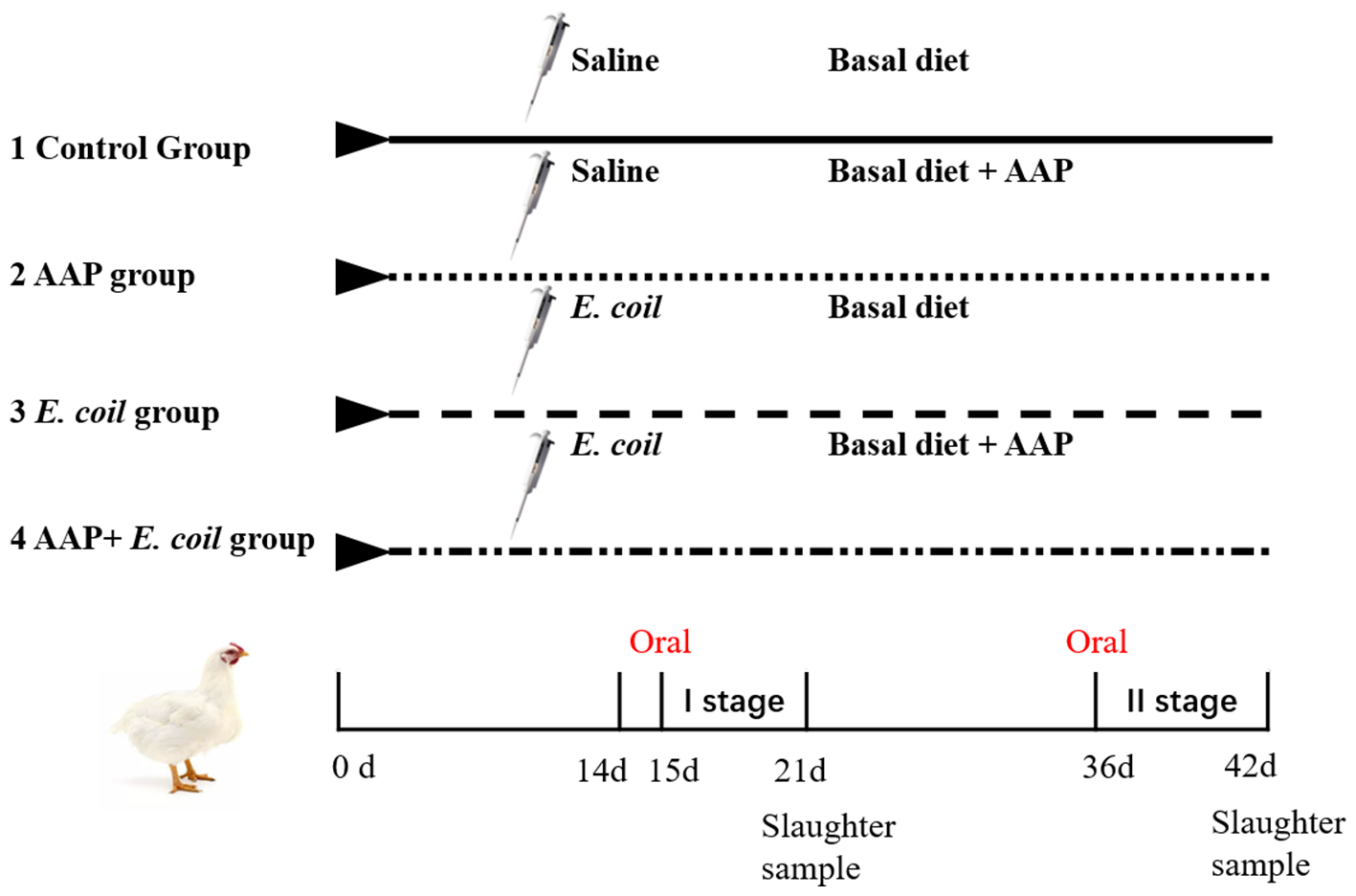
Flowchart of the broiler experiment. AAP *Artemisia annua* L. polysaccharide group, *E. coli Escherichia. coli* challenge group, AAP + *E. coli Artemisia annua* L. polysaccharide + *E. coli* challenge group.

A total of 256 Arbor Acres broilers were purchased from a commercial hatchery (Hohhot, China). These birds were randomly divided into four treatments according to their initial body weight including the control group (fed basal diet), the AAP group (fed basal diet supplemented with 750 mg/kg AAP), *E. coli* group (fed basal diet and orally administered *E. coli* (3.2 × 10^11^ CFU/kg body weight)), and AAP + *E. coli* group (fed basal diet supplemented with 750 mg/kg AAP and orally administered *E. coli* (3.2 × 10^11^ CFU/kg body weight)), respectively. Each treatment group had eight replicates with eight birds per replicate, half male and half female. The trial included the pre-feeding period (d 1 to 14), and the formal trial period (d 15 to 42). During d 15 to 20 (*E. coli* challenge phase I), the *E. coli* treatment groups were given oral administration of 0.5 mL *E. coli* saline suspension (2.5 × 10^11^ CFU/mL) in the morning, the control group was given the same amount of normal saline. During the earlier period (d 1 to 21), the chicks are characterized by an underdeveloped immune system and limited resistance, rendering them vulnerable to external pathogens. Additionally, their intestinal microflora is in the establishment stage, and is susceptible to environmental influence. This phase is provided an opportune time for the observation and analysis of the infection process, pathogenic mechanisms, and the broiler’s resistance to *E. coli*. As broilers progress into the growth period (d 22 to 42), their physiological and immune function gradually improve. This developmental stage presents an opportunity for deeper exploration of the infection characteristics and pathogenicity of *E. coli* at different growth stages. By conducting *E. coli* challenge experiments during this phase, valuable insights into the interplay between *E. coli* infection and broiler physiology can be gained, contributing to a more nuanced understanding of host-pathogen interactions in poultry production systems. Therefore, during d 36 to 42 (*E. coli* challenge phase II), the *E. coli* treatment groups were given oral administration of 2 mL *E. coli* saline suspension (3.2 × 10^11^ CFU/mL) in the morning, the control group was given the same amount of normal saline. By conducting *E. coli* challenges in two stages, the dynamics of *E. coli* infection in broilers can be comprehensively investigated, thus enhancing the understanding of its pathogenicity. The workflow is shown in Figure 1. Diets were formulated to meet the nutritional recommendations of the Feeding Standard of Chicken, China (NY/T 33-2004) (Table 1). The birds had *ad libitum* access to food and water. At the end of the experiment, eight birds were randomly selected from each treatment (one chicken per replicate) and slaughtered on d 21 and d 42, respectively. Blood was collected from wing veins, and the serum was separated and preserved at −20°C. Additionally, the jejunal tissues and chyme were promptly collected and stored at −80°C for further analysis.

**Table 1:** Composition and nutrient levels of the basal diet (as-fed basis), %.

Items	1 to 21 days of age	22 to 42 days of age
Ingredients		
Corn	52.50	58.80
Soybean meal	40.00	33.80
Soybean oil	3.00	3.00
Dicalcium phosphate	1.90	1.80
Limestone	1.08	1.22
Salt	0.37	0.37
Lysine	0.05	0.03
Methionine	0.19	0.07
Premix^a^	0.80	0.80
Choline	0.11	0.11
Total	100.0	100.0
Nutrient levels^b^		
Metabolic energy (MJ/kg)	12.42	12.62
Crude protein	21.77	19.65
Calcium	1.00	1.02
Available phosphorus	0.44	0.42
Lysine	1.34	1.15
Methionine	0.55	0.40
Cystine	0.40	0.36

a Premix provided the following per kilogram of diet: vitamin A 9000 IU, vitamin D_3_ 3,000 IU, vitamin E 26 mg, vitamin K_3_ 1.20 mg, vitamin B_1_ 3.00 mg, vitamin B_2_ 8.00 mg, vitamin B_6_ 4.40 mg, vitamin B_12_ 0.012 mg, nicotinic acid 45 mg, folic acid 0.75 mg, biotin 0.20 mg, calcium pantothenate 15 mg, Fe 100 mg, Cu 10 mg, Zn 108 mg, Mn 120 mg, I 1.5 mg, Se 0.35 mg.

b Crude protein was measured value, while others were all calculated values.

### 2.5 Growth performance and nutrient apparent metabolic rate

On d 1, 14, 21, 35, and 42 of the experiment, the body weight (BW) and feed intake of experiment birds in each replicate were meticulously measured and recorded. Subsequently, the average daily gain (ADG), average daily feed intake (ADFI), and feed conversion ratio (FCR) were calculated for each stage of the experiment. During d 19 to 21 and d 40 to 42 of the experiment, feces was methodically collected from each replicate, and the fecal weight and feed intake of each replicate were meticulously recorded after continuous fecal collection for three consecutive days. The apparent nutrient retention was measured via the total feces collection method, and then calculating the apparent metabolic rate of feed dry matter (DM), crude protein (CP), crude fat (ether extract, EE), calcium (Ca), and phosphorus (P). To provide a more accurate representation of growth, the values were corrected for mortality rate.

### 2.6 Sample collection

On d 21 and 42, one bird was randomly selected from each replicate pen and weighed accurately. Blood samples were then collected from the wing vein and centrifuged (3,000 × g, 10 min) at 4°C. The serum was then carefully harvested and stored at −20°C until further analysis. The birds were humanely euthanized by cervical dislocation. The jejunum’s middle segment was carefully excised, rinsed with sterile cold saline, placed in a sterile tube, and then flash-frozen in liquid nitrogen. The samples were stored at −80°C for mRNA expression analysis. Additionally, 1 cm section of the jejunum was gently washed with 0.9% (w/vol) physiological saline and fixed in a 10% formalin solution for further morphological examination.

### 2.7 Intestinal permeability

The serum diamine oxidase (DAO, product code: A088-1, assay range: 0 U/L–100 U/L) was tested using commercial assay kits, read absorbance OD at 340 nm, the difference in inter-assay is less 10% (Jiancheng Bioengineering Institute, Co. Ltd., Nanjing, China). The serum endotoxins (ET, catalogue number: JYM0109Ch, assay range: 1.5 ng/mL–100 ng/mL) and D-lactate (D-LA, catalogue number: JYM0160Ch, assay range: 0.8 ng/mL–50 ng/mL) were tested using ELISA kits, read absorbance OD at 450 nm, the difference in intra-assay and inter-assay is less than 9 and 15%, respectively, (Wuhan Gene Beauty Biotechnology Co. Ltd., China).

### 2.8 Preparation of intestinal homogenate

The jejunal tissues were processed using a hand-held homogenizer (FA6/10, FLUKO, Shanghai, China) at 4°C in ice-cold 0.9% NaCl solution (wt/vol, 1:9) and then centrifuged at 4000 × g for 15 min at 4°C. The resulting supernatant was collected for further analysis. The protein content of the homogenate was determined using the Coomassie Brilliant Blue assay (product code: A045-2, absorbance OD at 595 nm) according to the manufacturer’s instructions for the commercial kits (Nanjing Jiancheng Institute of Bioengineering, Nanjing, China).

### 2.9 Immune indexes in serum and tissue

The concentration of interleukin-1 beta (IL-1β, catalogue number: JYM0041Ch, assay range: 1.5 pg/mL–100 pg/mL), interleukin-6 (IL-6, catalogue number: JYM0028Ch, assay range: 1 pg/mL–100 pg/mL), tumor necrosis factor-α (TNF-α, catalogue number: JYM0033Ch, assay range: 1.2 pg/mL–100 pg/mL), immunoglobulin A (IgA, catalogue number: JYM0012Ch, assay range: 1 μg/mL–70 μg/mL), immunoglobulin G (IgG, catalogue number: JYM0001Ch, assay range: 0.3 μg/mL–20 μg/mL), immunoglobulin M (IgM, catalogue number: JYM0060Ch, assay range: 8 ng/mL–450 ng/mL), and secretory immunoglobulin A (sIgA, catalogue number: JYM0036Ch, assay range: 15 pg/mL–1000 pg/mL) in the serum and intestinal tissue homogenate supernatant was analyzed using ELISA kits (Wuhan Gene Beauty Biotechnology Co. Ltd., China) following the manufacturer’s instructions, read absorbance OD at 450 nm, the difference in intra-assay and inter-assay is less than 9 and 15%, respectively.

### 2.10 Antioxidant indexes in serum and tissue

The total antioxidant capacity (TAC, product code: A015-2-1, assay range: 0.5 mM–2 mM, absorbance OD at 405 nm), the activity of total superoxide dismutase (SOD, product code: A001-1, absorbance OD at 550 nm), glutathione peroxidase (GPx, product code: A005, absorbance OD at 412 nm), and catalase (CAT, product code: A007-1-1, absorbance OD at 405 nm), and the concentration of glutathione (GSH, product code: A006-2-1, absorbance OD at 405 nm) and malondialdehyde (MDA, product code: A003-1, absorbance OD at 532 nm) in the serum and intestinal tissue were determined by a spectrophotometric method according to the instructions of the commercial kits (Nanjing Jiancheng Institute of Bioengineering, Nanjing, China).

### 2.11 Intestinal morphology

A small portion of the jejunum tissues were fixed in 10% formalin and then embedded in paraffin, then sliced into thin sections with a thickness of 7 μm using a rotary microtome (YD-1508R Rotary Slicer, Yidi Medical Equipment Factory, Jinhua, Zhejiang, China), and stained with hematoxylin and eosin. The 10 intact villi of each tissue were precisely measured for villus height (VH) and crypt depth (CD) by high-resolution photography under 100× magnification using a light microscope (Olympus SZX10, Tokyo, Japan), and the average values for each tissue for each tissue were calculated.

### 2.12 Total RNA extraction and reverse transcription

Total RNA from jejunal tissue samples was obtained using Trizol reagent (TaKaRa Biotechnology Co. Ltd., Dalian, China). The purity and quantity of the total RNA were assessed with a spectrophotometer (Pultton P200CM, San Jose, CA, United States). Subsequently, the DNA of total RNA was removed by incubation for 2 min at 42°C with a gDNA digester (Yeasen Biotechnology Co., Ltd. Shanghai, China). Total RNA was reverse transcribed to cDNA on Labcycler (SensoQuest GmbH, Göttingen, Germany) using Hifair^®^ II SuperMix plus (Yeasen Biotechnology Co., Ltd. Shanghai, China). The reactions were incubated for 5 min at 85°C, 30 min at 42°C, and 5 min at 85°C.

### 2.13 Quantitative real-time PCR

Real-time PCR was performed using LightCycler^®^ 96 instrument and application software analysis system (LightCycler^®^ 96 Instrument, Roche Diagnostics, Indiana, United States) with a Hieff^®^ qPCR SYBR^®^ Green Master Mix (No Rox) Kit (Yeasen Biotechnology Co., Ltd. Shanghai, China). The reactions were: 95°C for 30 s (hold stage), followed by 40 cycles of 95°C for 5 s, 60°C for 30 s, and 72°C for 20 s (PCR stage), then 95°C for 15 s, 60°C for 1 min, 95°C for 15 s (melt-curve stage). All samples were run in duplicate in 10 μL reaction volume and melt curve analysis was performed to ensure the specificity of the PCR-amplified product. The mRNA expression of each gene was normalized to that of 𝛽-actin. The fold change relative to the control group was analyzed according to the 2^−ΔΔCT^ method. The specific sequences of primers are listed in Table 2.

**Table 2:** Sequences of primers for RT-qPCR.

Genes	Genbank ID	Primer sequence (5′ to 3’)	Length, bp
*Claudin-1*	NM_001013611.2	F: GGTATGGCAACAGAGTGGCT	91
		R: CAGCCAATGAAGAGGGCTGA	
*Claudin-3*	NM 204202.1	F: CTTCATCGGCAACAACATCGTGAC	113
		R: CCAGCATGGAGTCGTACACCTTG	
*Occludin*	NM_205128.1	F: ATCGCCTCCATCGTCTACATC	90
		R: GCTGCACATGGCCAACAAG	
*ZO-1*	XM_015278981.1	F: TATGCACAAGGAGGTCAGCC	97
		R: TTGGCCGAAGCATTCCATCT	
*JAM-2*	NM_001006257	F: AGCCTCAAATGGGATTGGATT	59
		R: CATCAACTTGCATTCGCTTCA	
*Mucin-2*	NM_001318434	F: AAATGTATCTGTCGCCCCTCA	121
		R: TGTCGCCATCCTTTATTGTTG	
*IL-1β*	NM_204524	F: CAGCCTCAGCGAAGAGACCTT	84
		R: ACTGTGGTGTGCTCAGAATCC	
*IL-6*	HM179640	F: AAATCCCTCCTCGCCAATCT	106
		R: CCCTCACGGTCTTCTCCATAAA	
*TNF-α*	NM_204267	F: TGTGTATGTGCAGCAACCCGTAGT	229
		R: GGCATTGCAATTTGGACAGAAGT	
*TLR4*	NM_001030693	F: TTCAGAACGGACTCTTGAGTGG	131
		R: CAACCGAATAGTGGTGACGTTG	
*MyD88*	NM_001030962	F: CCTGGCTGTGCCTTCGGA	198
		R: TCACCAAGTGCTGGATGCTA	
*NF-κB p65*	D13721	F: CAGCCCATCTATGACAACCG	151
		R: CAGCCCAGAAACGAACCTC	
*CAT*	NM_001031215.1	F: GTTGGCGGTAGGAGTCTGGTCT	182
		R: GTGGTCAAGGCATCTGGCTTCTG	
*SOD*	NM_205064.1	F: TTGTCTGATGGAGATCATGGCTTC	98
		R: TGCTTGCCTTCAGGATTAAAGTGA	
*GPx*	NM_001163245.1	F: CAAAGTTGCGGTCAGTGGA	136
		R: AGAGTCCCAGGCCTTTACTACTTTC	
*Nrf2*	NM_205117.1	F: GATGTCACCCTGCCCTTAG	215
		R. CTGCCACCATGTTATTCC	
*Keap1*	XM_015274015.1	F: TGCCCCTGTGGTCAAAGTG	104
		R: GGTTCGGTTACCGTCCTGC	
*β-Actin*	NM_205518	F: GCCAACAGAGAGAAGATGACAC	118
		R: GTAACACCATCACCAGAGTCCA	

F, forward primer; R, reverse primer; ZO-1, zonula occludens-1; JAM-2, junctional adhesion molecule-2; IL-1β, interleukin 1 beta; IL-6, interleukin 6; TNF-α, tumor necrosis factor α; TLR4, toll like receptor 4; MyD88, myeloid differentiation primary response 88; NF-κB p65, nuclear factor kappa B p65; CAT, catalase; SOD, superoxide dismutase; GPx, glutathione peroxidase; Nrf2, nuclear factor erythroid-2-related factor 2; Keap1, kelch like ECH associated protein 1; β-Actin, beta-actin.

### 2.14 16S rRNA amplicon sequencing and bioinformatics

Microbial community genomic DNA was extracted from jejunum content samples using the E.Z.N.A.^®^ soil DNA Kit (Omega Bio-tek, Norcross, GA, United States) according to manufacturer’s instructions. The DNA extract was checked on 1% agarose gel, and DNA concentration and purity were determined with NanoDrop 2000 UV–vis spectrophotometer (Thermo Scientific, Wilmington, United States). The hypervariable region V3-V4 of the bacterial 16S rRNA gene were amplified with primer pairs 338F (5’-ACTCCTACGGGAGGCAGCAG-3′) and 806R (5’-GGACTACHVGGGTWTCTAAT-3′) by an ABI GeneAmp^®^ 9,700 PCR thermocycler (ABI, CA, United States). The PCR amplification of 16S rRNA gene was performed as follows: initial denaturation at 95°C for 3 min, followed by 27 cycles of denaturing at 95°C for 30 s, annealing at 55°C for 30 s and extension at 72°Cfor 45 s, and single extension at 72°C for 10 min, and end at 10°C. The PCR mixtures contain 5 × Pro Taq 10 μL, forward primer (5 μM) 0.8 μL, reverse primer (5 μM) 0.8 μL, template DNA 10 ng/μL, and finally ddH_2_O up to 20 μL. PCR reactions were performed in triplicate. The PCR product was extracted from 2% agarose gel and purified using the AxyPrep DNA Gel Extraction Kit (Axygen Biosciences, Union City, CA, United States) according to manufacturer’s instructions and quantified using Quantus™ Fluorometer (Promega, United States). Purified amplicons were pooled in equimolar and paired-end sequenced on an Illumina MiSeq PE300 platform/NovaSeq PE250 platform (Illumina, San Diego, United States) according to the standard protocols by Majorbio Bio-Pharm Technology Co. Ltd. (Shanghai, China). The raw 16S rRNA gene sequencing reads were demultiplexed, quality-filtered by fastp version 0.20.0 and merged by Flash version 1.2.7. Operational taxonomic units (OTUs) with 97% similarity cutoff were clustered using Uparse version 7.1, and chimeric sequences were identified and removed. The taxonomy of each OTU representative sequence was analyzed by RDP Classifier version 2.2 against the 16S rRNA database (Silva v138) using confidence threshold of 0.7. α diversity, β diversity, community composition, and analysis of different communities are carried out on the I-Sanger Cloud Platform provided by Majorbio Bio-Pharm Technology Co., Ltd. (Qiao et al., 2022b).

### 2.15 Statistical analysis

Data were analyzed by one-way ANOVA with the general linear model procedure of SAS version 9.2 (SAS Institute Inc., Cary, NC), and a pen of broilers (a replicate) served as the experimental unit for all data. The differences among treatments were tested by Duncan’s multiple comparison analysis and were considered significant at *p* < 0.05. The results were expressed as the mean and standard error of the mean (SEM).

## 3 Results

### 3.1 Growth performance and nutrient apparent metabolic rate

As shown in Table 3, compared with the control group, dietary AAP significantly increased BW of broilers on d 42 (*p* < 0.01). *E. coli*-challenged broilers had significantly decreased BW on d 21, d 35, and d 42, ADG and ADFI on d 36–42 (*p* < 0.05). And there was no difference between AAP + *E. coli* group and control group. In addition, dietary AAP supplementation significantly enhanced ADG compared to the control group during d 36–42 (*p* < 0.01). However, there was no difference in FCR among treatment groups.

**Table 3:** Effects of AAP on growth performance of broilers challenged by *E. coli*.

Items	Treatment				SEM	*p*-value
	CON	AAP	*E.coli*	AAP + *E.coli*		
BW, g						
d 14	396.08	392.42	392.54	396.00	8.08	0.978
d 21	695.19^a^	704.56^a^	632.55^b^	656.67^ab^	17.21	0.033
d 35	1687.09^ab^	1718.93^a^	1576.46^c^	1608.57^bc^	28.94	0.008
d 42	2239.05^b^	2412.8^a^	2079.01^c^	2127.62^bc^	46.60	0.001
ADG, g						
d 15–21	49.10^a^	52.63^a^	40.38^b^	46.35^ab^	2.11	0.009
d 36–42	78.60^b^	83.60^a^	72.95^c^	74.59^bc^	1.36	0.001
ADFI, g						
d 15–21	68.60	70.17	62.51	68.51	5.42	0.824
d 36–42	171.14^b^	183.19^a^	165.79^b^	167.58^b^	2.20	0.001
FCR						
d 15–21	1.35	1.29	1.69	1.42	0.12	0.118
d 36–42	2.28	2.16	2.28	2.28	0.06	0.434

CON, control group; AAP, *Artemisia annua* L. polysaccharide group; *E. coli, Escherichia. coli* group; AAP +* E. coli, Artemisia annua* L. polysaccharide + *Escherichia. coli* group; BW, body weight; ADG, average daily gain; ADFI, average daily feed intake; FCR, feed conversion ratios. ^a,b,c^Different superscripts within the same row indicate a significant difference (*p* < 0.05).

**Figure 2:**
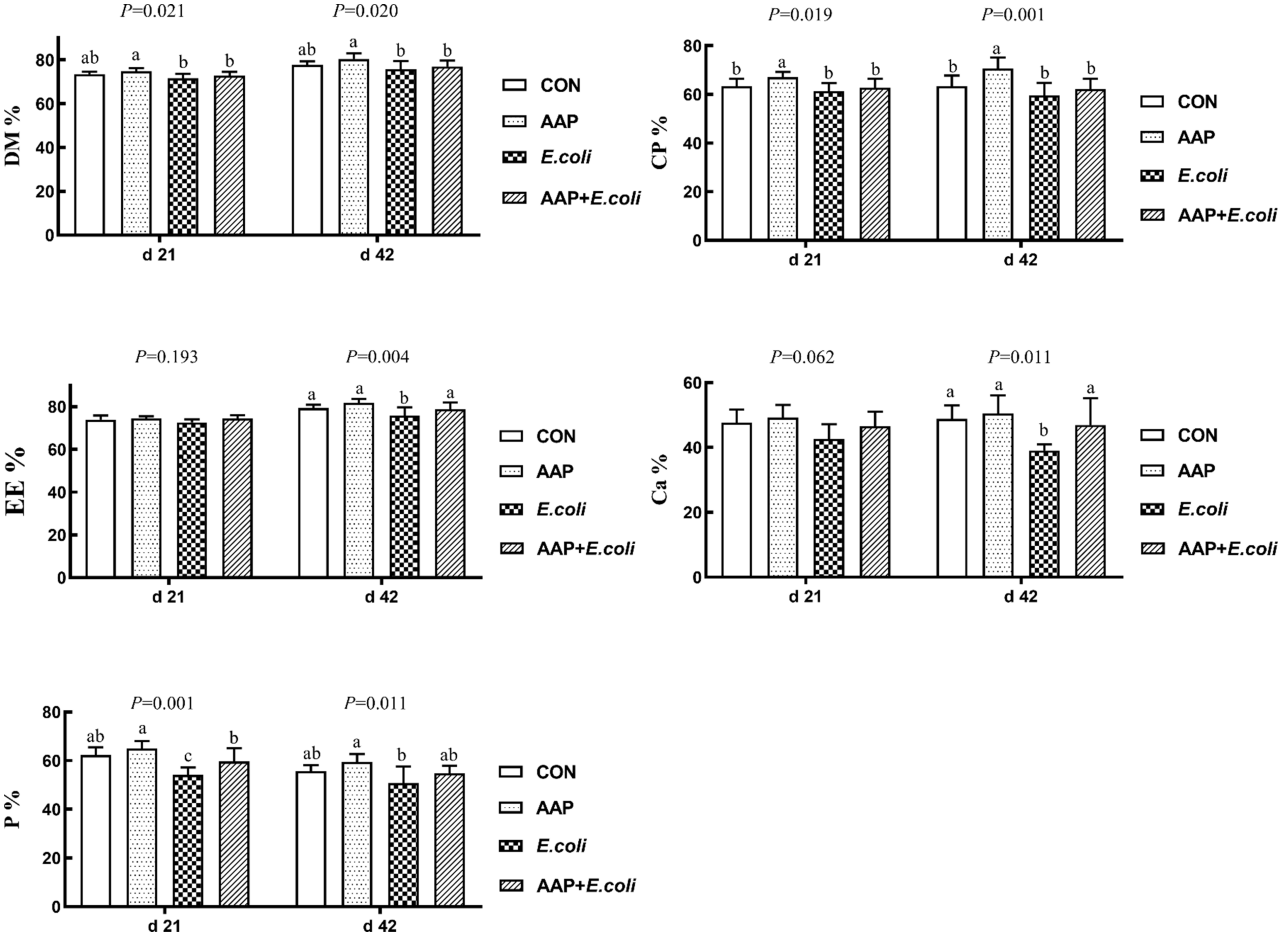
Effects of AAP on apparent nutrient metabolic rate of broilers challenged by *E. coli*. CON control group, AAP *Artemisia annua* L. polysaccharide group, *E. coli Escherichia. coli* group, AAP + *E. coli Artemisia annua* L. polysaccharide + *Escherichia. coli* group, DM dry matter, CP crude protein, EE ether extract, Ca calcium, P phosphorus. ^a,b,c^Different letters in the same period indicate significant differences between groups (*p* < 0.05).

As described in Figure 2, compared with the control group, dietary AAP supplementation significantly increased the apparent metabolic rate of CP on d 21 and 42 (*p* < 0.05). The apparent metabolic rate of P on d 21, EE and Ca on d 42 in *E. coli*-challenged broilers was significantly lower than that of control group (*p* < 0.05). And there was no difference between AAP + *E. coli* group and the control group.

### 3.2 Intestinal permeability

As summarized in Table 4, compared with the control group, *E. coli*-challenged broilers had significantly increased DAO activity on d 21 and 42, and ET content on d 42 (*p* < 0.05), and there was no difference between the AAP + *E. coli* group and the control group.

**Table 4:** Effect of AAP on intestinal permeability indicators of broilers challenged by *E. coli*.

Items	Treatment				SEM	*p*-value
	CON	AAP	*E.coli*	AAP + *E.coli*		
d 21						
D-LA, ng/mL	8.94^ab^	8.28^b^	9.82^a^	9.12^ab^	0.28	0.025
DAO, U/L	56.21^bc^	42.77^c^	91.56^a^	75.18^ab^	6.20	0.001
ET, ng/mL	15.39	14.90	17.17	15.93	0.58	0.099
d 42						
D-LA, ng/mL	9.01^ab^	8.12^b^	9.99^a^	9.29^ab^	0.38	0.049
DAO, U/L	62.77^b^	50.57^b^	82.56^a^	65.67^ab^	5.15	0.015
ET, ng/mL	16.78^b^	15.97^b^	21.21^a^	17.13^b^	0.78	0.003

CON, control group; AAP, *Artemisia annua* L. polysaccharide group; *E. coli, Escherichia. coli* group; AAP +* E. coli, Artemisia annua* L. polysaccharide + *Escherichia. coli* group; DAO, diamine oxidase; D-LA, D-lactate; ET, endotoxin. ^a,b,c^Different superscripts within the same row indicate a significant difference (*p* < 0.05).

### 3.3 Intestinal morphology

**Figure 3:**
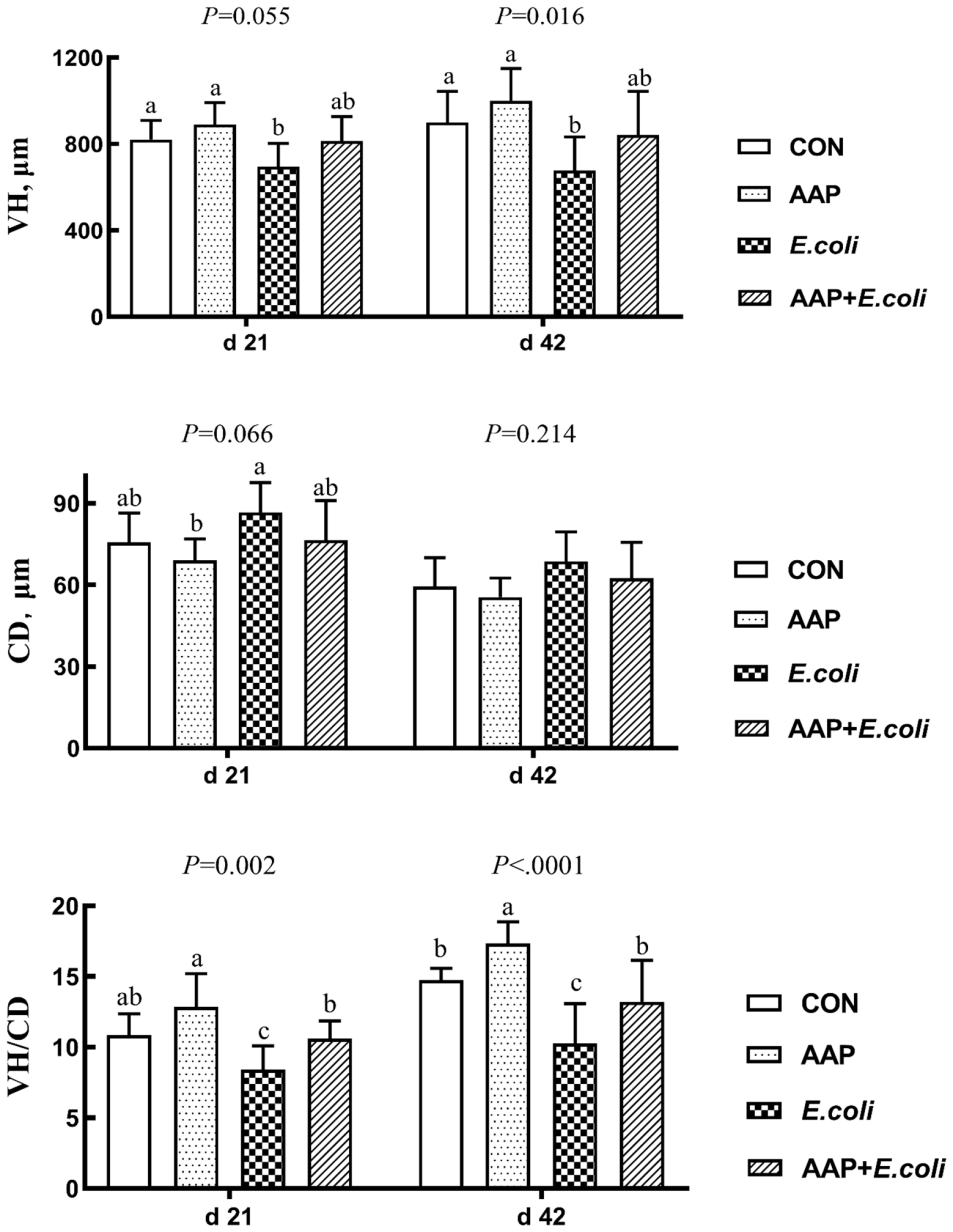
Effects of AAP on intestinal morphology of broilers challenged by *E. coli*. CON control group, AAP *Artemisia annua* L. polysaccharide group, *E. coli Escherichia. coli* group, AAP + *E. coli Artemisia annua* L. polysaccharide + *Escherichia. coli* group, VH villus height, CD crypt depth, VH/CD villus height to crypt depth ratio. ^a–c^Different letters in the same period indicate significant differences between groups (*p* < 0.05).

As illustrated in Figure 3, compared with the control group, *E. coli*-challenged broilers had reduced VH on d 21 (*p* = 0.055) and 42 (*p* < 0.05), and VH/CD d 21 and 42 (*p* < 0.01), but there was no difference between the AAP + *E. coli* group and the control group. Moreover, dietary AAP supplementation significantly enhanced VH/CD on d 42 (*p* < 0.01).

### 3.4 Immune indexes in serum and tissue

As shown in Table 5, dietary AAP supplementation significantly increased serum IgA content on d 21 (*p* < 0.01). Compared with the control group, *E. coli*-challenged broilers significantly reduced serum IgM content on d 21 (*p* < 0.05) and increased IL-6 content on d 42 (*p* < 0.01), and there was no difference between the AAP + *E. coli* group and the control group.

**Table 5:** Effect of AAP on serum immune indicators in broilers challenged by *E. coli*.

Items	Treatment				SEM	*p*-value
	CON	AAP	*E.coli*	AAP + *E.coli*		
d 21						
IgA, μg/mL	68.37^b^	82.67^a^	61.48^b^	68.85^b^	2.69	0.001
IgG, μg/mL	16.29	15.27	16.17	15.99	0.71	0.785
IgM, ng/mL	394.79^a^	403.34^a^	326.07^b^	393.84^a^	15.24	0.011
IL-1β, pg/mL	79.75	77.08	76.17	78.01	3.77	0.933
IL-6, pg/mL	85.59	78.70	85.67	78.70	3.39	0.295
TNF-α, pg/mL	79.76	77.39	84.15	77.56	2.63	0.413
d 42						
IgA, μg/mL	67.55	78.93	66.95	89.04	5.74	0.124
IgG, μg/mL	14.30	14.96	16.43	15.47	0.58	0.179
IgM, ng/mL	369.85	376.92	404.20	353.10	14.17	0.214
IL-1β, pg/mL	101.40^ab^	89.21^b^	132.58^a^	126.70^a^	10.34	0.027
IL-6, pg/mL	81.20^b^	77.38^b^	95.73^a^	80.82^b^	3.08	0.006
TNF-α, pg/mL	78.80	81.67	85.27	83.40	5.14	0.876

CON, control group; AAP, *Artemisia annua* L. polysaccharide group; *E. coli, Escherichia. coli* group; AAP +  *E. coli, Artemisia annua* L. polysaccharide + *Escherichia. coli* group; IgA, immunoglobulin; IgG, immunoglobulin G; IgM, immunoglobulin M; IL-1β, interleukin-1 beita; IL-6, interleukin-6; TNF-α, tumor necrosis factor α. ^a,b^Different superscripts within the same row indicate a significant difference (*p* < 0.05).

As presented in Table 6, compared with the control group, *E. coli*-challenged broilers had significantly decreased jejunum IgG content (*p* < 0.05), but there was no difference between the AAP + *E. coli* group and the control group on d 21. The jejunum IgM content of *E. coli* group and AAP + *E. coli* group was significantly lower than that of the control group on d 21 (*p* < 0.01). Compared with the control group, *E. coli*-challenged broilers markedly increased jejunum IL-1β and IL-6 content (*p* < 0.05), but there was no difference between the AAP + *E. coli* group and the control group on d 42.

**Table 6:** Effects of AAP on intestinal immune indicators in broilers challenged by *E. coli*.

Items	Treatment				SEM	*p*-value
	CON	AAP	*E.coli*	AAP + *E.coli*		
d 21						
IgG, μg/mg prot.	8.00^a^	8.42^a^	5.93^b^	7.27^ab^	0.55	0.040
IgM, ng/mg prot.	187.74^a^	179.05^a^	118.35^b^	137.99^b^	10.67	0.002
sIgA, pg/mg prot.	350.84	383.17	309.62	330.05	25.99	0.399
IL-1β, pg/mg prot.	30.60^a^	24.64^b^	29.78^a^	27.21^ab^	1.20	0.046
IL-6, pg/mg prot.	39.93	32.58	37.94	37.64	1.62	0.102
TNF-α, pg/mg prot.	39.84^a^	31.01^b^	38.58^a^	33.81^ab^	2.05	0.050
d 42						
IgG, μg/mg prot.	8.14	9.21	7.66	8.64	0.42	0.157
IgM, ng/mg prot.	169.40	186.55	141.89	157.79	11.09	0.147
sIgA, pg/mg prot.	320.09	365.34	342.69	378.28	14.92	0.111
IL-1β, pg/mg prot.	29.53^b^	28.77^b^	35.98^a^	32.28^ab^	1.43	0.017
IL-6, pg/mg prot.	38.86^b^	36.84^b^	47.10^a^	42.84^ab^	1.83	0.010
TNF-α, pg/mg prot.	32.86	29.10	37.50	32.93	2.04	0.127

CON, control group; AAP, *Artemisia annua* L. polysaccharide group; *E. coli, Escherichia. coli* group; AAP +   *E. coli, Artemisia annua* L. polysaccharide + *Escherichia. coli* group; IgG, immunoglobulin G; IgM, immunoglobulin M; sIgA, secretory immunoglobulin A; IL-1β, interleukin-1 beita; IL-6, interleukin-6; TNF-α, tumor necrosis factor α. ^a,b^Different superscripts within the same row indicate a significant difference (*p* < 0.05).

### 3.5 Antioxidant indexes in serum and tissue

As shown in Table 7, compared with the control group, *E. coli*-challenged broilers had significantly reduced serum GPx activity on d 21 (*p* < 0.05), but there was no difference between the AAP + *E. coli* group and the control group. Compared with the control group, dietary AAP inclusion had notably decreased serum MDA concentration on d 21 (*p* < 0.01).

**Table 7:** Effect of AAP on serum antioxidant indicators in broilers challenged by *E. coli*.

Items	Treatment				SEM	*p*-value
	CON	AAP	*E.coli*	AAP + *E.coli*		
d 21						
TAC, mM	0.82	0.88	0.76	0.82	0.04	0.483
CAT, U/mL	1.80	2.04	1.61	1.80	0.18	0.494
SOD, U/mL	152.73	166.33	122.41	141.57	11.11	0.111
GPx, U/mL	1304.8^ab^	1535.2^a^	879.9^c^	1003.5^bc^	118.48	0.011
GSH, μmol/L	31.06	35.43	24.53	27.83	2.75	0.150
MDA, nmol/mL	3.45^ab^	2.95^c^	3.61^a^	3.14^bc^	0.10	0.003
d 42						
TAC, mM	0.72	0.78	0.72	0.72	0.04	0.749
CAT, U/mL	1.11^ab^	1.34^a^	0.77^b^	0.99^b^	0.10	0.014
SOD, U/mL	171.27	190.58	157.06	172.41	13.74	0.533
GPx, U/mL	2662.4^ab^	2986.7^a^	2153.6^b^	2696.5^ab^	156.62	0.021
GSH, μmol/L	29.16^ab^	38.95^a^	25.18^b^	28.12^b^	2.96	0.058
MDA, nmol/mL	3.47	3.20	3.90	3.51	0.23	0.298

CON, control group; AAP, *Artemisia annua* L. polysaccharide group; *E. coli, Escherichia. coli* group; AAP +   *E. coli, Artemisia annua* L. polysaccharide +  *Escherichia. coli* group; TAC, total antioxidant capacity; CAT, catalase; SOD, total superoxide dismutase; GPx, glutathione peroxidase; GSH, glutathione; MDA, malondialdehyde. ^a,b,c^Different superscripts within the same row indicate a significant difference (*p* < 0.05).

As presented in Table 8, compared with the control group, *E. coli*-challenged broilers had significantly reduced jejunum TAC, CAT, SOD activity (*p* < 0.05), but there was no difference between the AAP + *E. coli* group and the control group on d 42. Moreover, the jejunum MDA content of *E. coli*-challenged broilers trended to be higher than that of the control group on d 21 (*p* = 0.098) and 42 (*p* = 0.052).

**Table 8:** Effects of AAP on intestinal antioxidant indicators in broilers challenged by *E. coli*.

Items	Treatment				SEM	*p*-value
	CON	AAP	*E.coli*	AAP + *E.coli*		
d 21						
TAC, μmol/g prot.	48.57^ab^	57.03^a^	39.06^b^	44.05^b^	2.69	0.004
CAT, U/mg prot.	1.08^ab^	1.31^a^	0.80^b^	1.01^b^	0.09	0.011
SOD, U/mg prot.	279.30	307.32	237.48	273.71	17.43	0.152
GPx, U/mg prot.	9.76^ab^	11.66^a^	7.73^b^	8.81^b^	0.63	0.011
GSH, μmol/g prot.	42.56^ab^	47.99^a^	30.20^b^	41.45^ab^	3.84	0.068
MDA, nmol/mg prot.	0.93^b^	0.912^b^	1.28^a^	1.02^ab^	0.10	0.098
d 42						
TAC, μmol/g prot.	118.14^ab^	132.87^a^	86.38^c^	100.55^bc^	5.82	<0.001
CAT, U/mg prot.	1.15^a^	1.32^a^	0.83^b^	1.15^a^	0.08	0.013
SOD, U/mg prot.	287.02^ab^	305.28^a^	243.51^c^	265.79^bc^	9.68	0.005
GPx, U/mg prot.	12.48^ab^	14.96^a^	9.94^b^	11.88^ab^	1.09	0.056
GSH, μmol/g prot.	72.11^ab^	80.10^a^	58.76^b^	61.12^b^	5.08	0.038
MDA, nmol/mg prot.	1.02^b^	0.90^b^	1.36^a^	1.08^ab^	0.09	0.052

CON, control group; AAP, *Artemisia annua* L. polysaccharide group; *E. coli, Escherichia. coli* group; AAP +  *E. coli, Artemisia annua* L. polysaccharide +  *Escherichia. coli* group; TAC, total antioxidant capacity; CAT, catalase; SOD, total superoxide dismutase; GPx, glutathione peroxidase; GSH, glutathione; MDA, malondialdehyde. ^a,b,c^Different superscripts within the same row indicate a significant difference (*p* < 0.05).

### 3.6 Intestinal tight junction protein-related mRNA expression

**Figure 4:**
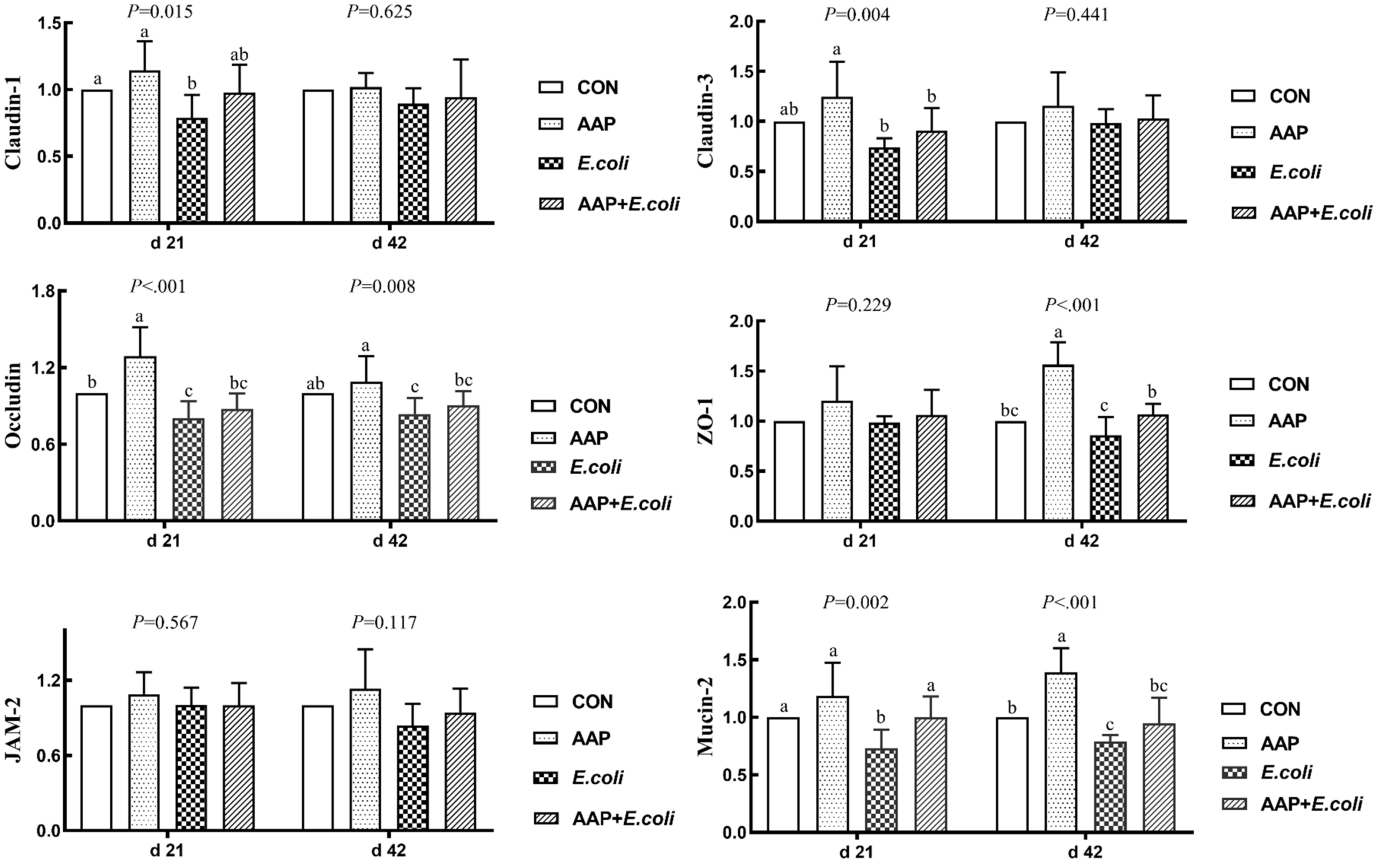
Effects of AAP on tight junction protein mRNA expression level of broilers challenged by *E. coli*. CON control group, AAP *Artemisia annua* L. polysaccharide group, *E. coli Escherichia. coli* group, AAP + *E. coli Artemisia annua* L. polysaccharide + *Escherichia. coli* group, *ZO-1* zonula occludens-1, *JAM-2* junctional adhesion molecule-2. ^a–c^Different letters in the same period indicate significant differences between groups (*p* < 0.05).

As shown in Figure 4, compared with the control group, dietary AAP supplementation extremely significantly increased jejunum mRNA expression levels of *Occludin* (d 21), *ZO-1* (d 42) and *Mucin-2* (d 42) (*p* < 0.01). Besides, compared with the control group, *E. coli*-challenged broilers noticeably reduced jejunum mRNA expression level of *Claudin-1* (d 21), *Occludin* (d 21 and 42), *ZO-1* (d 42) and *Mucin-2* (d 21 and 42) (*p* < 0.05), but there was no difference between AAP + *E. coli* group and control group.

### 3.7 Intestinal proinflammatory factor-related mRNA expression

**Figure 5:**
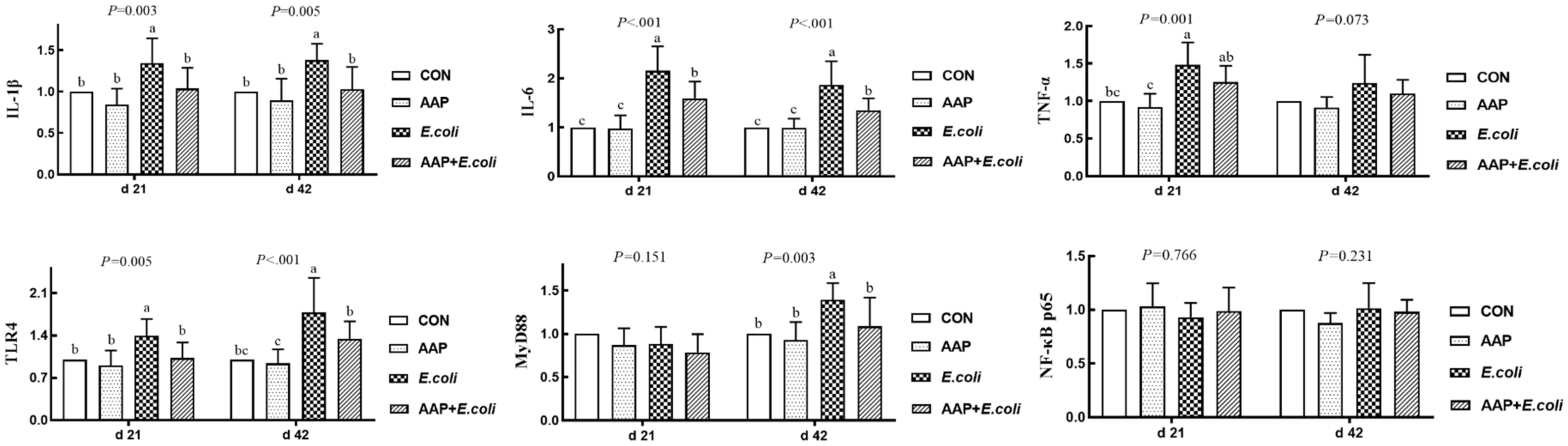
Effects of AAP on inflammatory-related mRNA expression level of broilers challenged by *E. coli*. CON control group, AAP *Artemisia annua* L. polysaccharide group, *E. coli Escherichia. coli* group, AAP + *E. coli Artemisia annua* L. polysaccharide + *Escherichia. coli* group, *IL-1β* interleukin 1 beta, *IL-6* interleukin 6, *TNF-α* tumor necrosis factor α, *TLR4* toll like receptor 4, *MyD88* myeloid differentiation primary response 88, *NF-κB p65* nuclear factor kappa B p65. ^a–c^Different letters in the same period indicate significant differences between groups (*p* < 0.05).

As indicated in Figure 5, compared with the control group, *E. coli*-challenged broilers had extremely significantly increased jejunum mRNA expression level of *IL-1β* and *TLR4* (*p* < 0.01), while there was no difference between the AAP + *E. coli* group and the control group on d 21 and 42. Compared with the control group, *E. coli* group and AAP + *E. coli* group significantly increased jejunum mRNA expression level of *IL-6*, but which was markedly lower in AAP + *E. coli* group than that in the *E. coli* group on d 21 and 42 (*p* < 0.01). Compared with the control group, *E. coli*-challenged broilers extremely significantly increased jejunum mRNA expression level of *TNF-α* (d 21) and *MyD88* (d 42) (*p* < 0.01), but there was no difference between the AAP + *E. coli* group and the control group.

### 3.8 Intestinal antioxidant-related mRNA expression

**Figure 6:**
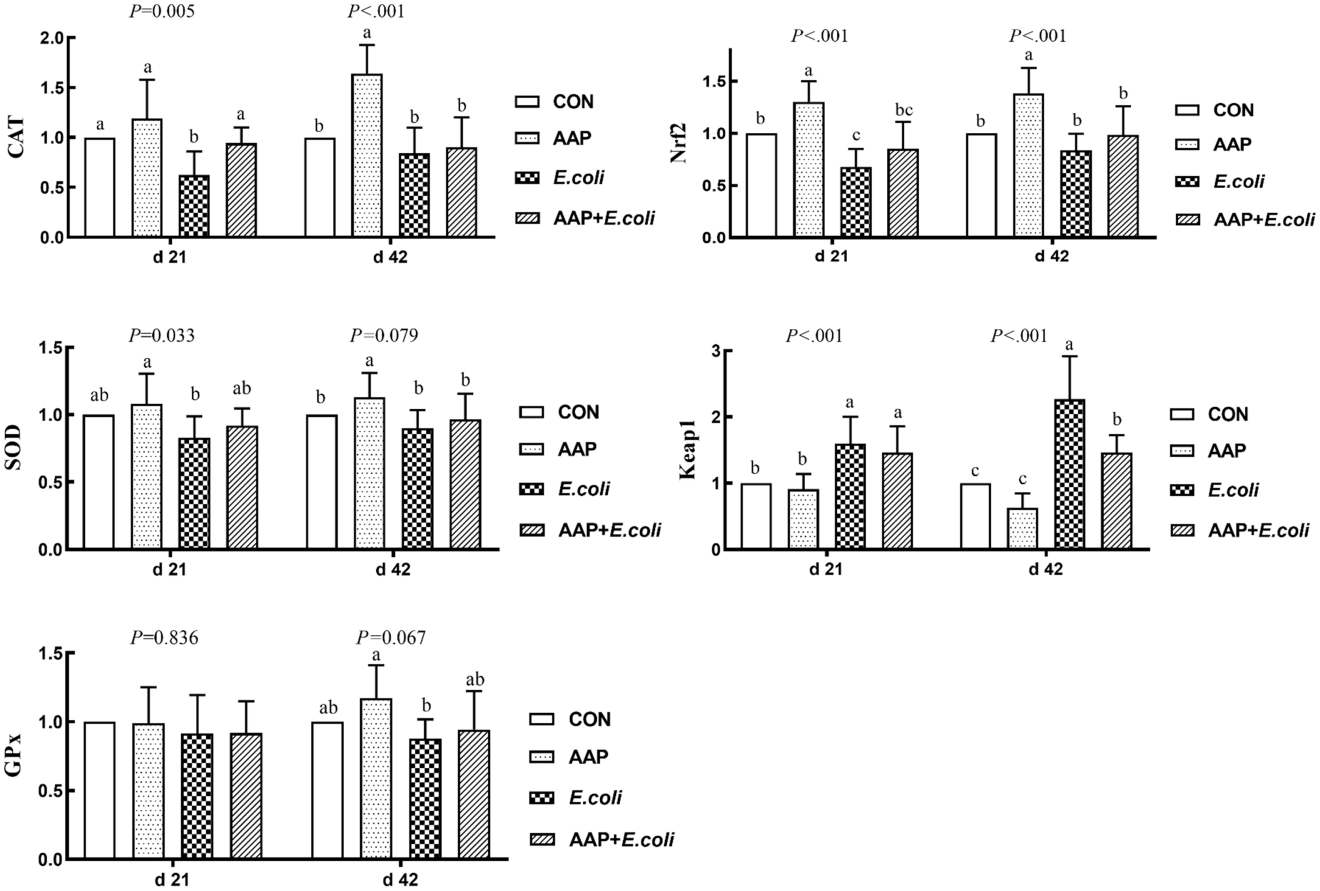
Effects of AAP on antioxidant-related mRNA expression level of broilers challenged by *E. coli*. CON control group, AAP *Artemisia annua* L. polysaccharide group, *E. coli Escherichia. coli* group, AAP + *E. coli Artemisia annua* L. polysaccharide + *Escherichia. coli* group, *CAT* catalase, *SOD* total superoxide dismutase, *GPx* glutathione peroxidase, *Nrf2* nuclear factor erythroid-2-related factor 2, *Keap1* kelch like ECH associated protein 1. ^a–c^Different letters in the same period indicate significant differences between groups (*p* < 0.05).

As shown in Figure 6, compared with the control group, *E. coli*-challenged broilers dramatically decreased jejunum mRNA expression level of *CAT* and *Nrf2* (*p* < 0.01), but there was no difference between the AAP + *E. coli* group and the control group on d 21. Dietary AAP supplementation extremely significantly increased jejunum mRNA expression level of *CAT* (d 42), *SOD* (d 42; *p* = 0.079) and *Nrf2* (d 21 and 42) (*p* < 0.01). Besides, compared with the control and AAP group, *E. coli* group and AAP + *E. coli* group markedly significantly increased the jejunum mRNA expression level of *Keap1* on d 21 and 42 (*p* < 0.01), however, AAP + *E. coli* group had significantly lower jejunum mRNA expression level of *Keap1* than *E. coli* group on d 42 (*p* < 0.01).

### 3.9 Intestinal microbial analysis

**Figure 7:**
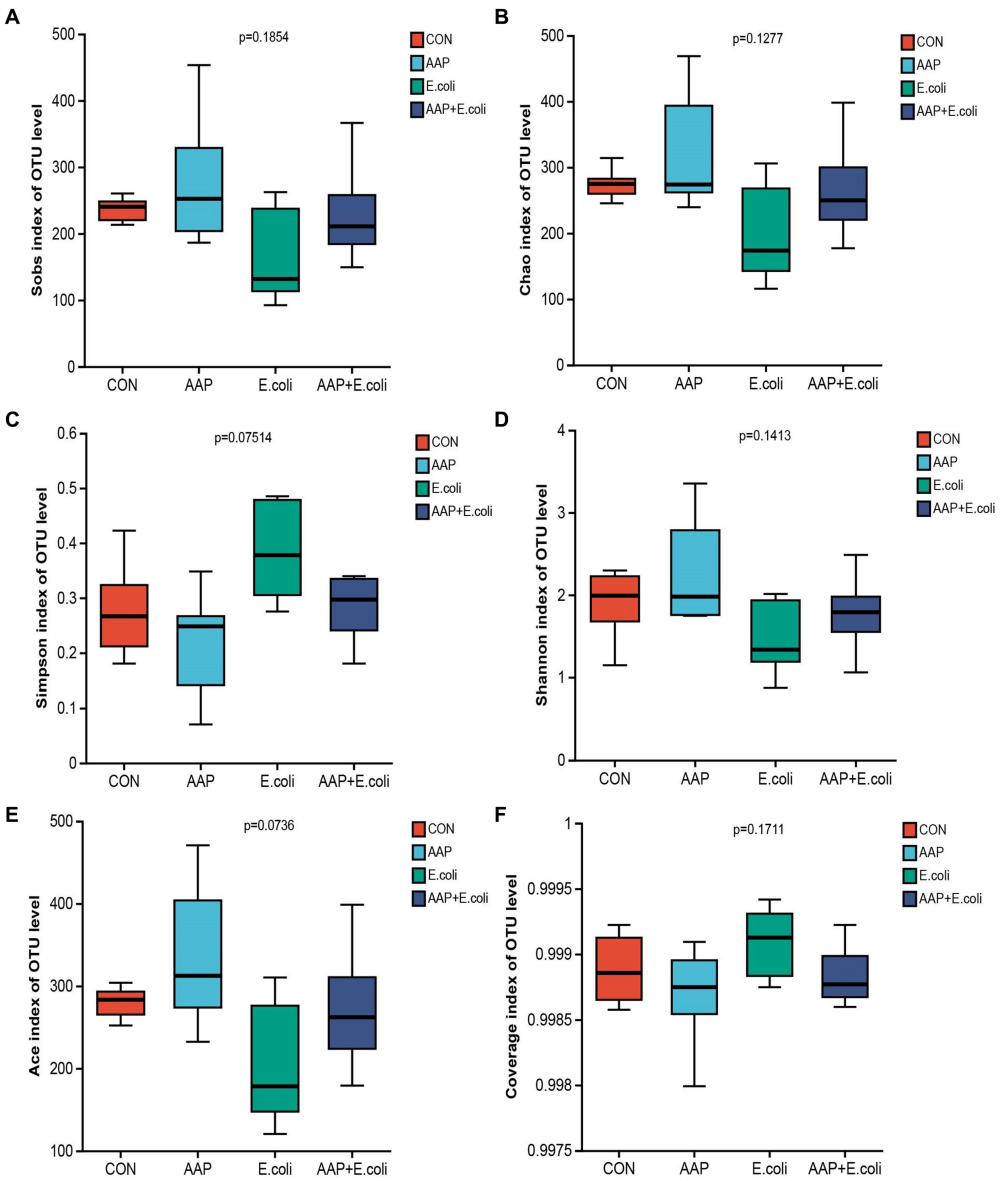
Effects of AAP on α diversity index of jejunal microbiota of broilers on d 21 challenged by *E. coli*. CON control group, AAP *Artemisia annua* L. polysaccharide group, *E. coli Escherichia. coli* group, AAP + *E. coli Artemisia annua* L. polysaccharide + *Escherichia. coli* group. Sobs index, Chao index, Simpson index, Shannon index, Ace index, Coverage index **(A–F)**.

**Figure 8:**
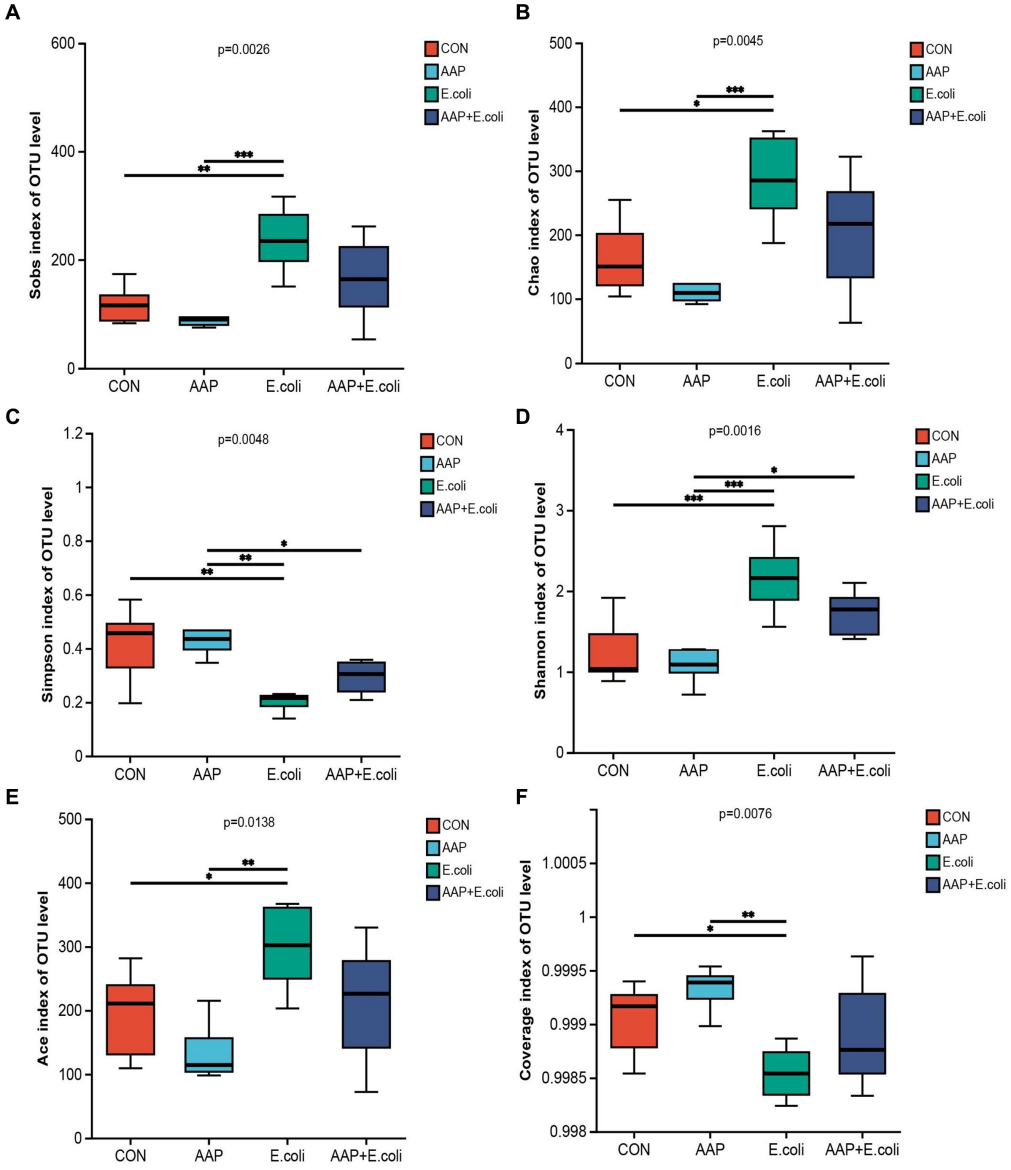
Effects of AAP on α diversity index of jejunal microbiota of broilers on d 42 challenged by *E. coli*. CON control group, AAP *Artemisia annua* L. polysaccharide group, *E. coli Escherichia. coli* group, AAP + *E. coli Artemisia annua* L. polysaccharide + *Escherichia. coli* group. Sobs index, Chao index, Simpson index, Shannon index, Ace index, Coverage index **(A–F)**. Symbol “*,” “**,” “***” indicates the significant difference based on *p* < 0.05, *p* < 0.01, *p* < 0.001.

The bacterial α diversity indices are presented in Figures 7, 8. There was no significant difference in α diversity indices (Sobs, Chao, Simpson, Shannon, Ace, and Coverage) on d 21 (Figures 7A–F; *p* > 0.05). However, the Simpson indexes (Figure 7C; *p* = 0.075) and Ace indexes (Figure 7E; *p* = 0.074) of the *E. coli* group tended to be significantly higher and lower, respectively, than those of the other groups. On d 42, *E. coli*-challenged broilers significantly increased the indexes of Sobs, Chao, Shannon and Ace (Figures 8A,B,D,E; *p* < 0.05), and significantly decreased the indexes of Simpon and Coverage (Figures 8C,F; *p* < 0.05). However, there was no difference between the AAP + *E. coli* group and the control group.

**Figure 9:**
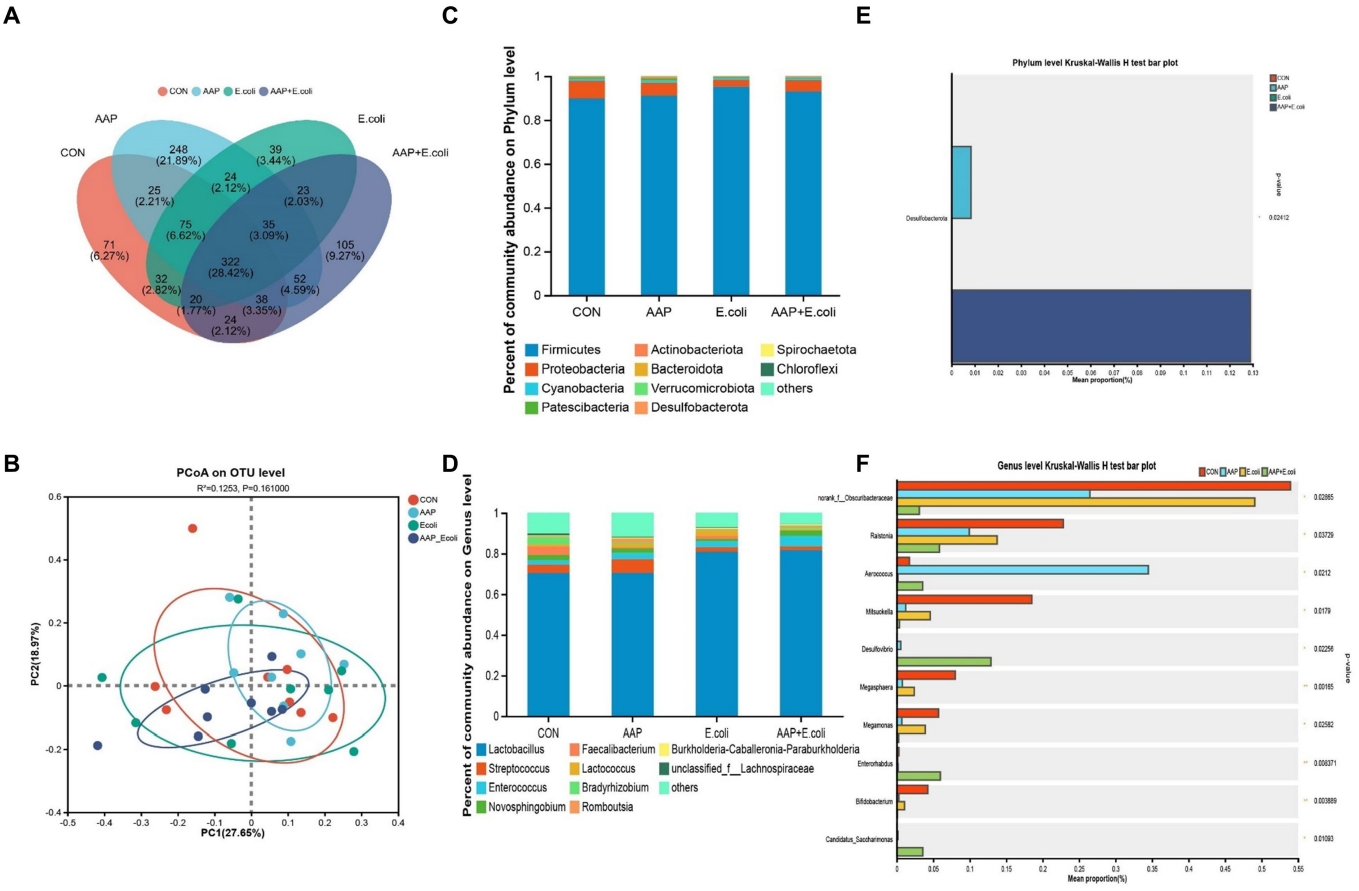
Effects of AAP on microbial composition of jejunal microbiota of broilers on d 21 challenged by *E. coli*. CON control group, AAP *Artemisia annua* L. polysaccharide group, *E. coli Escherichia. coli* group, AAP + *E. coli Artemisia annua* L. polysaccharide + *Escherichia. coli* group. Venn diagram based on OTU **(A)**. Principal coordinate analysis (PCoA) plot **(B)**. Jejunal microbiota composition at the phylum level, and alterations of the abundance of bacterial phylum found in the jejunum of broilers **(C,D)**. Jejunal microbiota composition at the genus level, and alterations of the abundance of bacterial genus found in the jejunum of broilers **(E,F)**.

On d 21, the Venn diagram (Figure 9A) showed that a total of 322 OTU is shared among the 4 treatment groups. In addition, the unique OUT numbers corresponding to the control group, AAP group, *E. coli* group, and AAP + *E. coli* group were, respectively, 71, 248, 39, and 105 on d 21. Principal co-ordinates analysis (PCoA) (Figure 9B) showed that the microbial community composition changed among the four treatment groups on d 21. The composition of the jejunal microbiota is shown in Figure 9 on d 21. At the phylum level, the dominant bacteria were *Firmicutes*, *Proteobacteria*, *Cyanobacteria*, *Patescibacteria*, and *Actinobacteria* (Figure 9C). The abundance of *Desulfobacterota* in phylum level in AAP group and AAP + *E. coli* group was significantly higher than that in control group and *E. coli* group (Figure 9D; *p* < 0.05). At the genus level, the dominant bacteria were *Lactobacillus*, *Streptococcus*, *Enterococcus*, *Novosphingobium*, and *Romboutsia* (Figure 9E). Compared with the control group and *E. coli* group, the AAP group and AAP + *E. coli* group significantly decreased the abundance of *norank_f_Obscuribacteraceae*, *Ralstonia*, *Mitsuokella*, *Megasphaera*, *Megamonas* and *Bifidobacterium*, and significantly increased the abundance of *Aerococcus*, *Desulfovibrio and Candidatus_Saccharimonas* at the genus level (Figure 9F; *p* < 0.05). Besides, the abundance of *Enterorhabdus* in the AAP + *E. coli* group was significantly higher than that in the other groups (Figure 9F; *p* < 0.05).

**Figure 10:**
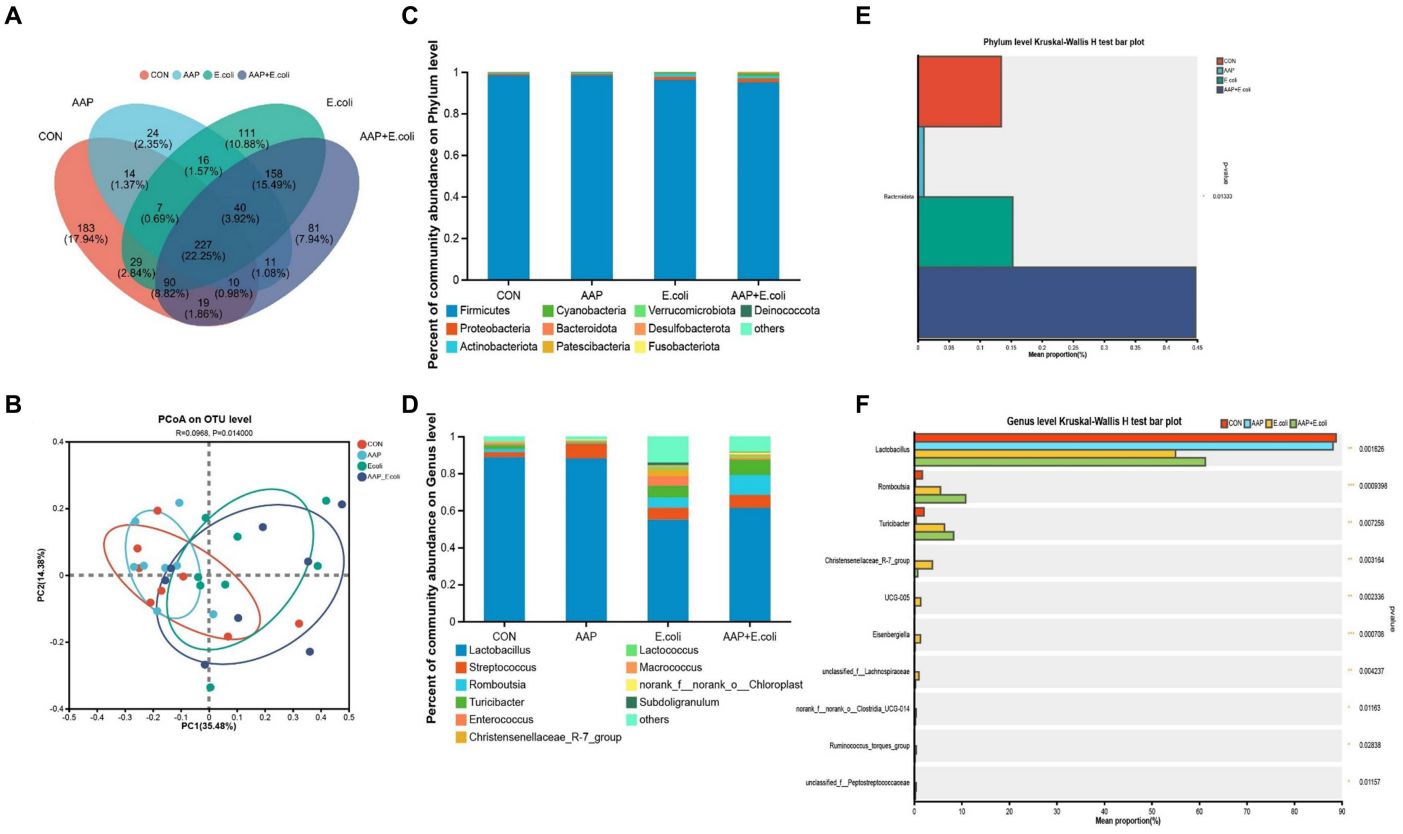
Effects of AAP on microbial composition of jejunal microbiota of broilers on d 42 challenged by *E. coli*. CON control group, AAP *Artemisia annua* L. polysaccharide group, *E. coli Escherichia. coli* group, AAP + *E. coli Artemisia annua* L. polysaccharide + *Escherichia. coli* group. Venn diagram based on OTU **(A)**. Principal coordinate analysis (PCoA) plot **(B)**. Jejunal microbiota composition at the phylum level, and alterations of the abundance of bacterial phylum found in the jejunum of broilers **(C,D)**. Jejunal microbiota composition at the genus level, and alterations of the abundance of bacterial genus found in the jejunum of broilers **(E,F)**.

On d 42, the Venn diagram (Figure 10A) showed that a total of 227 OTU is shared among the 4 treatment groups. In addition, the unique OUT numbers corresponding to the control group, AAP group, *E. coli* group, and AAP + *E. coli* group were, respectively, 183, 24, 111, and 81 on d 42. Principal co-ordinates analysis (PCoA) (Figure 10B) showed that the microbial community composition changed dramatically among the four treatment groups on d 42. The composition of the jejunal microbiota is shown in Figure 10 on d 42. At the phylum level, the dominant bacteria were *Firmicutes*, *Proteobacteria*, *Actinobacteria*, *Cyanobacteria*, and *Bacteroidota* (Figure 10C). The abundance of *Bacteroidota* in AAP + *E. coli* group was significantly higher than the other treatment groups at the phylum level (Figure 10D; *p* < 0.05). At the genus level, the dominant bacteria were *Lactobacillus*, *Streptococcus*, *Enterococcus*, *Lactococcus*, and *Turicibacter* (Figure 10E). Compared with the control group and AAP group, the *E. coli* group and AAP + *E. coli* group significantly decreased the abundance of *Lactobacillus*, and significantly increased the abundance of *Romboutsia*, *Turicibacter*, *Christensenellaceae_R-7_group*, *UCG-005*, *Eisenbergiella*, *unclassified_f_Lachnospiraceae*, *norank_f_norank_o_Clostridia_UCG-014*, *Ruminococcus_torques_group* and *unclassified_f_Peptostreptococcaceae* at the genus level (Figure 10F; *p* < 0.05). Among them, the abundance of *Christensenellaceae_R-7_group*, *UCG-005*, *Eisenbergiella*, *unclassified_f_Lachnospiraceae*, *norank_f_norank_o_Clostridia_UCG-014*, *Ruminococcus_torques_group* and *unclassified_f_Peptostreptococcaceae* in AAP + *E. coli* group was significantly higher than that in *E. coli* group (Figure 10F; *p* < 0.05).

**Figure 11:**
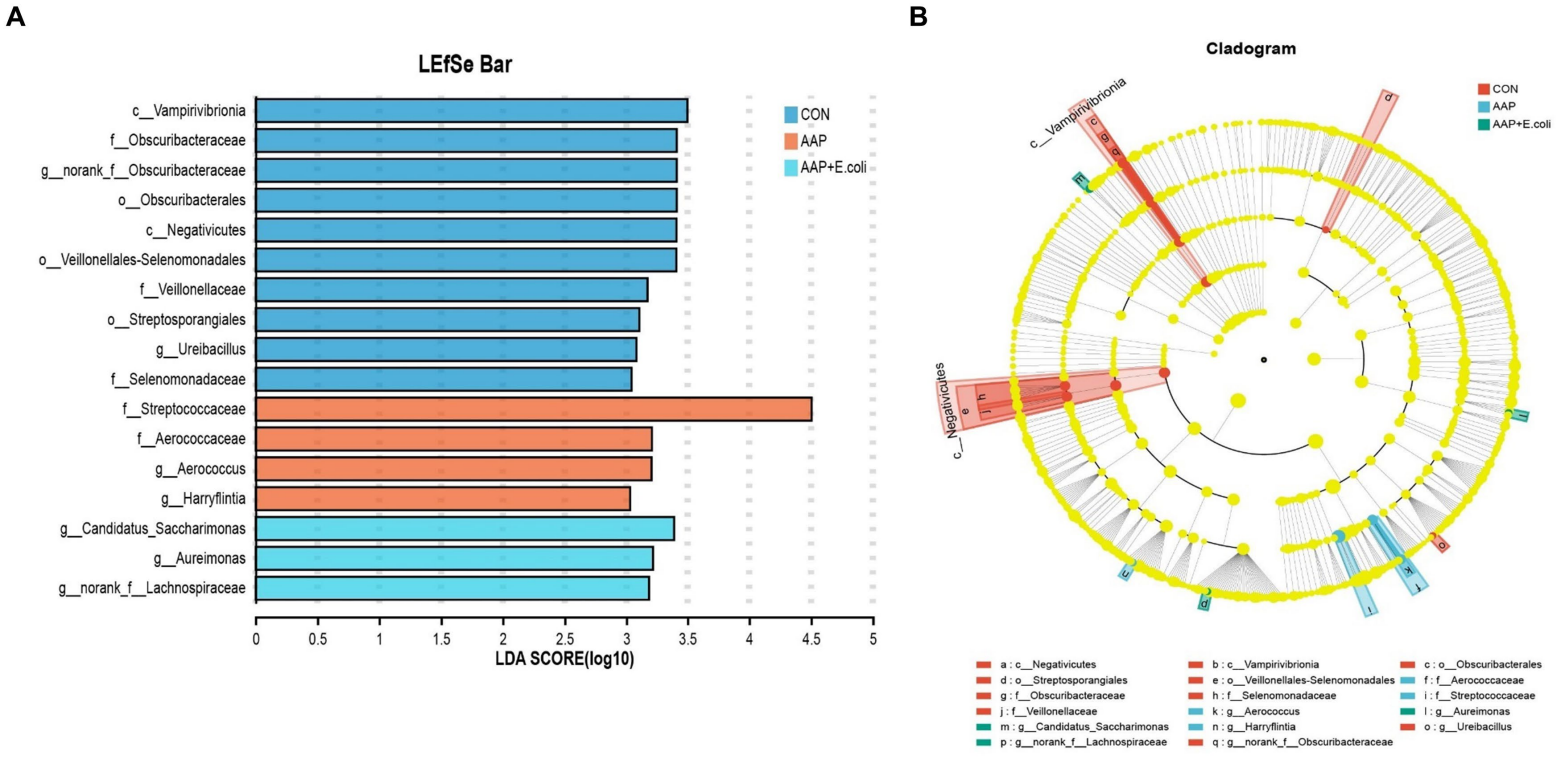
Linear discriminant analysis (LDA) effect size (LEfSe) analysis of jejunal microbiota on d 21. CON control group, AAP *Artemisia annua* L. polysaccharide group, *E. coli Escherichia. coli* group, AAP + *E. coli Artemisia annua* L. polysaccharide + *Escherichia. coli* group. LDA bar chart **(A)**. LDA cladogram **(B)**. LDA scores generated for the differentially abundant microbiota (LDA > 3, *p* < 0.05).

**Figure 12:**
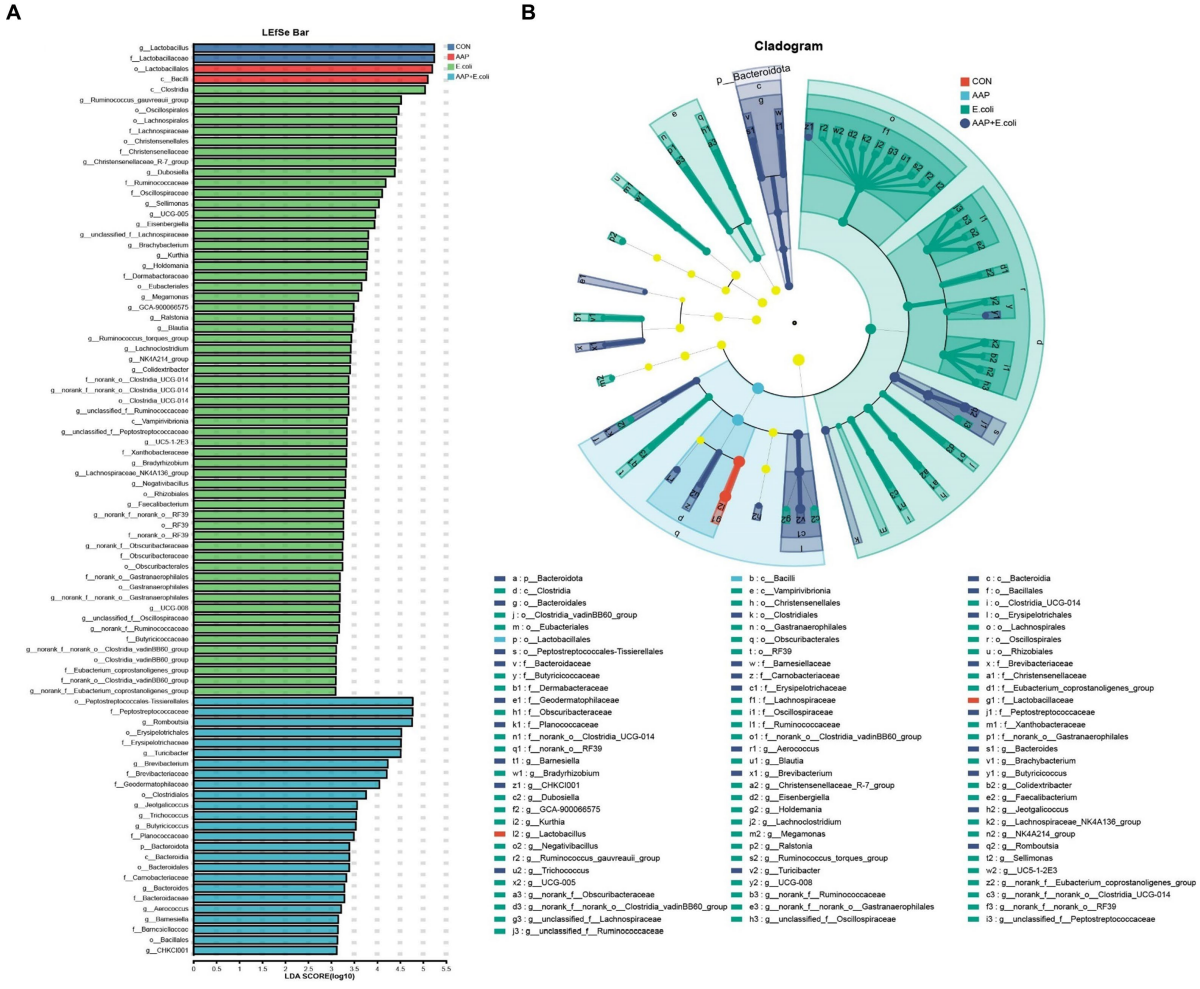
Linear discriminant analysis (LDA) effect size (LEfSe) analysis of jejunal microbiota on d 42. CON control group, AAP *Artemisia annua* L. polysaccharide group, *E. coli Escherichia. coli* group, AAP + *E. coli Artemisia annua* L. polysaccharide + *Escherichia. coli* group. LDA bar chart **(A)**. LDA cladogram **(B)**. LDA scores generated for the differentially abundant microbiota (LDA > 3, *p* < 0.05).

The linear discriminant analysis (LDA = 3) effect size (LEfSe) algorithm was used to analyze the taxonomic abundance of microbiota. The results for d 21 are shown in Figures 11A,B. *c_Vampirivibrionia*, *f_Obscuribacteraceae*, *g_norank_f_Obscuribacteraceae*, *o_Obscuribacterales*, *c_Negativicutes*, *o_Veillonellales-Selenomonadales*, *f_Veillonellaceae*, *o_Streptosporangiales*, *g_Ureibacillus*, and *f_Selenomonadaceae* were enhanced in the control group. *f_Streptococcaceae*, *f_Aerococcaceae, g_Aerococcus*, and *g_Harryflintia* were enhanced in AAP group. *g_Candidatus_Saccharimonas*, *g_Aureimonas*, and *g_norank_f_Lachnospiraceae* were enhanced in AAP + *E. coli* group. The results for d 42 are shown in Figures 12A,B. *g_Lactobacillus* and *f_Lactobacillaceae* were enhanced in the control group. *o_Lactobacillales* and *c_Bacilli* were enhanced in AAP group. *c_Clostridia, g_Ruminococcus_gauvreauii_group, o_Oscillospirales, o_Lachnospirales, f_Lachnospiraceae, o_Christensenellales, f_Christensenellaceae, g_Christensenellaceae_R-7_group, g_Dubosiella, f_Ruminococcaceae, f_Oscillospiraceae, g_Sellimonas, g_UCG-005*, and *g_Eisenbergiella* were enhanced in *E. coli* group (LDA ≥ 4). *o_Peptostreptococcales-Tissierellales, f_Peptostreptococcaceae, g_Romboutsia, o_Erysipelotrichales, f_Erysipelotrichaceae, g_Turicibacter, g_Brevibacterium, f_Brevibacteriaceae*, and *f_Geodermatophilaceae* were enhanced in AAP + *E. coli* group (LDA ≥ 4).

**Figure 13:**
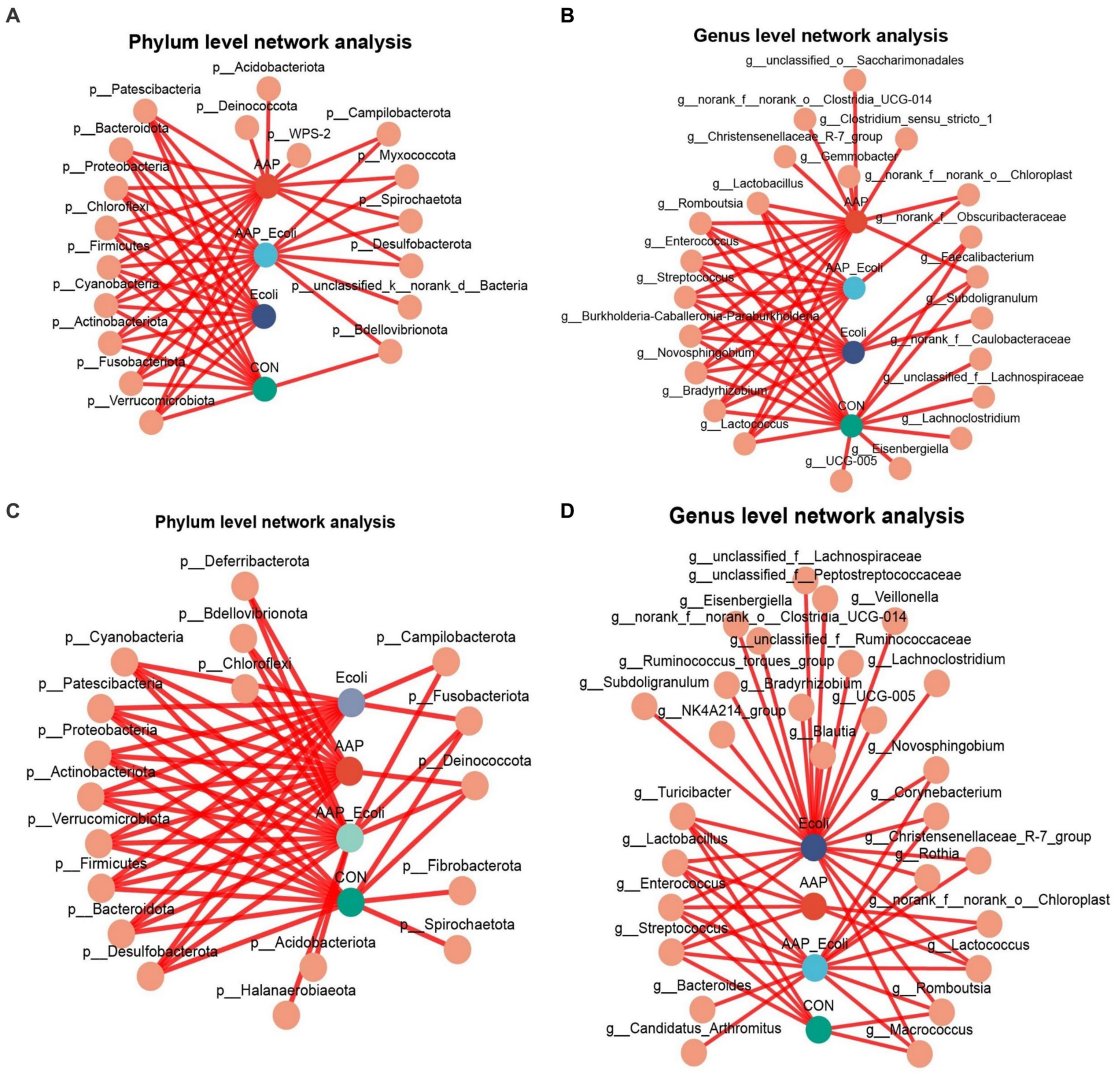
Co-occurrence network analysis of jejunal microbiota community at phylum and genus level. CON control group, AAP *Artemisia annua* L. polysaccharide group, *E. coli Escherichia. coli* group, AAP + *E. coli Artemisia annua* L. polysaccharide + *Escherichia. coli* group. d 21 phylum and genus level **(A,B)**. d 42 phylum and genus level **(C,D)**.

The co-occurrence network diagram of intestinal microbial at phylum and genus levels is depicted in Figure 13. The results for d 21 are shown in Figures 13A,B. At the phylum level, *p_Acidobacteriota*, *p_Deinococcota*, and *p_WPS-2* were only enriched in the AAP group (Figure 13A). *p_unclassified_k_norank_d_Bacteria* were only enriched in the AAP + *E. coli* group (Figure 13A). At the genus level, *g_norank_f_Caulobacteraceae, g_unclassified_f_Lachnospiraceae, g_Lachnoclostridium, g_Eisenbergiella*, and *g_UCG-005* were only enriched in the control group (Figure 13B). *g_Christensenellaceae_R-7_group, g_Clostridium_sensu_stricto_1, g_Gemmobacter, g_norank_f_norank_o_Clostridia_UCG-014*, and *g_unclassified_o_Saccharimonadales* were only enriched in the AAP group (Figure 13B). *g_Subdoilgranulum* were only enriched in the *E. coli* group (Figure 13B). The results for d 42 are shown in Figures 13C,D. At the phylum level, *p_Fibrobacterota* and *p_Spirochaetota* were only enriched in the control group (Figure 13C). *p_Acidobacteriota and p_Halanaerobiaeota* were only enriched in the AAP + *E. coli* group (Figure 13C). At the genus level, *g_bacteroides* and *g_Candidatus_Arthromitus* were only enriched in the AAP + *E. coli* group (Figure 13D). *g_Blautia, g_Bradyrhizobium, g_Lachnoclostridium, g_UCG-005, g_NK4A214_group, g_Subdoligranulum, g_Veillonella, g_Eienbergiella, g_Ruminococcus_torques_group, g_unclassified_f_Ruminococcaceae, g_unclassified_f_Lachnospiraceae, g_unclassified_f_Peptostreptococcaceae*, and *g_norank_f_norank_o_Clostridia_UCG-014* were only enriched in the *E. coli* group (Figure 13D).

### 3.10 Association between intestinal microbiota and oxidative stress status, and inflammatory markers

**Figure 14:**
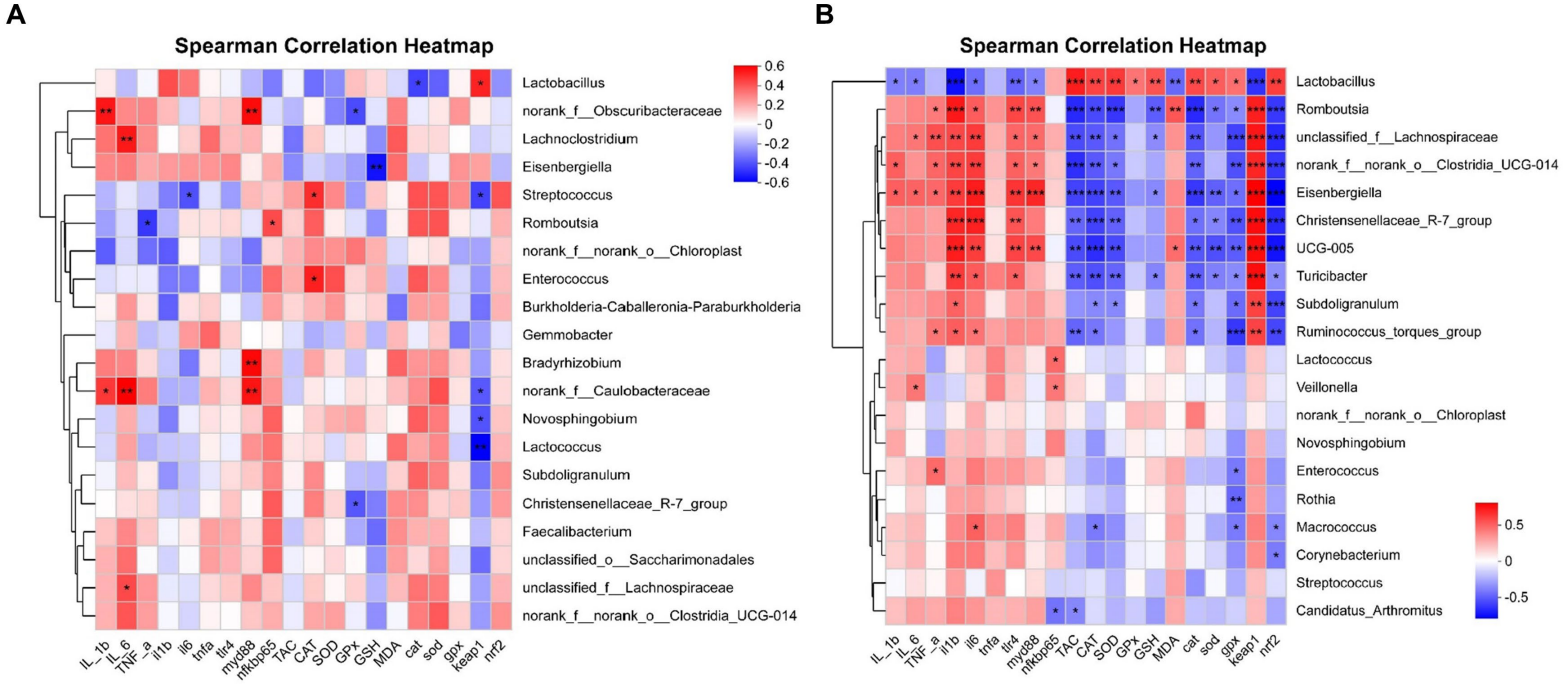
Correlation analysis of jejunal microbial proportions at the genus level with intestinal oxidative status and pro/anti-inflammatory markers among four groups, red represents a positive correlation, while blue represents a negative correlation. IL_1b interleukin-1 beita content, IL_6 interleukin-6 content, TNF-a tumor necrosis factor α content, il1b interleukin 1 beta gene expression, il6 interleukin 6 gene expression, tnfa tumor necrosis factor α gene expression, tlr4 toll like receptor 4 mRNA, myd88 myeloid differentiation primary response 88 gene expression, nfkbp65 nuclear factor kappa B p65 gene expression, TAC total antioxidant capacity, CAT catalase activity, SOD total superoxide dismutase activity, GPx glutathione peroxidase activity, GSH glutathione content, MDA malondialdehyde content, cat catalase gene expression, sod total superoxide dismutase gene expression, gpx glutathione peroxidase gene expression, nrf2 nuclear factor erythroid-2-related factor 2 gene expression, keap1 kelch like ECH associated protein 1 gene expression. *p < 0.05, **p < 0.01, and *** *p* < 0.001 indicate a significant correlation d 21 **(A)** and d 42 **(B)**.

The correlation between intestinal microbial proportions at the genus level and intestinal oxidative status, as well as pro/anti-inflammatory markers among four groups, is illustrated in Figure 14. The results for d 21 are shown in Figure 14A. The proportion of *Lactobacillus* had a positive correlation with *Keap1* mRNA expression level (*p* < 0.05), and had a negative correlation with *CAT* mRNA expression level (*p* < 0.05). The proportion of *norank_f_Obscuribacteraceae* had a positive correlation with IL-1β content and *MyD88* mRNA expression level (*p* < 0.01), and had a negative correlation with GPx activity (*p* < 0.05). The proportion of *Lachnoclostridium* had a positive correlation with IL-6 content (*p* < 0.01). The proportion of *Eisenbergiella* had a negative correlation with GSH content (*p* < 0.01). The proportion of *Streptococcus* had a positive correlation with CAT activity (*p* < 0.05), and had a negative correlation with *IL-6* and *Keap1* mRNA expression level (*p* < 0.05). The proportion of *Romboutsia* had a positive correlation with *NF-κB p65* mRNA expression level (*p* < 0.05), and had a negative correlation with TNF-α content (*p* < 0.05). The proportion of *Enterococcus* had a positive correlation with CAT activity (*p* < 0.05). The proportion of *Bradyrhizobium* had a positive correlation with *MyD88* mRNA expression level (*p* < 0.01). The proportion of *norank_f_Caulobacteraceae* had a positive correlation with IL-1β and IL-6 content, *MyD88* mRNA expression level (*p* < 0.01), and had a negative correlation with *Keap1* mRNA expression level (*p* < 0.05). The proportion of *Novosphingobium* and *Lactococcus* had a negative correlation with *Keap1* mRNA expression level (*p* < 0.05). The proportion of *Christensenellaceae_R-7_group* had a negative correlation with GPx activity (*p* < 0.05). The proportion of *unclassified_f_Lachnospiraceae* had a positive correlation with IL-6 content (*p* < 0.05).

The results for d 42 are shown in Figure 14B. The proportion of *Lactobacillus* had a positive correlation with TAC, CAT, SOD, and GPx activity, GSH content, *CAT*, *SOD*, *GPx*, and *Nrf2* mRNA expression level (*p* < 0.05), and had a negative correlation with IL-1β, IL-6 and MDA content, *IL-1β*, *IL-6*, *TLR4*, *MyD88*, and *Keap1* mRNA expression level (*p* < 0.05). The proportion of *Romboutsia* had a positive correlation with TNF-α and MDA content, and *IL-1β*, *IL-6*, *TLR4*, *MyD88*, and *Keap1* mRNA expression level (*p* < 0.05), and had a negative correlation with TAC, CAT, and SOD activity, GSH content, *CAT*, *SOD*, *GPx*, and *Nrf2* mRNA expression level (*p* < 0.05). The proportion of *unclassified_f_Lachnospiraceae* had a positive correlation with IL-6 and TNF-α content, and *IL-1β, IL-6, TLR4, MyD88*, and *Keap1* mRNA expression level (*p* < 0.05), and had a negative correlation with TAC, CAT, and SOD activity, GSH content, *CAT, GPx*, and *Nrf2* mRNA expression level (*p* < 0.05). The proportion of *norank_f_norank_o_Clostridia_UCG-014* had a positive correlation with IL-1β and TNF-α content, and *IL-1β, IL-6, TLR4, MyD88*, and *Keap1* mRNA expression level (*p* < 0.05), and had a negative correlation with TAC, CAT, and SOD activity, *CAT, GPx*, and *Nrf2* mRNA expression level (*p* < 0.05). The proportion of *Eisenbergiella* had a positive correlation with IL-1β, IL-6 and TNF-α content, and *IL-1β, IL-6, TLR4, MyD88*, and *Keap1* mRNA expression level (*p* < 0.05), and had a negative correlation with TAC, CAT, and SOD activity, GSH content, *CAT, SOD, GPx*, and *Nrf2* mRNA expression level (*p* < 0.05). The proportion of *Christensenellaceae_R-7_group* had a positive correlation with *IL-1β, IL-6, TLR4*, and *Keap1* mRNA expression level (*p* < 0.05), and had a negative correlation with TAC, CAT, and SOD activity, *CAT, SOD, GPx*, and *Nrf2* mRNA expression level (*p* < 0.05). The proportion of *UCG-005* had a positive correlation with MDA content, and *IL-1β, IL-6, TLR4, MyD88*, and *Keap1* mRNA expression level (*p* < 0.05), and had a negative correlation with TAC, CAT, and SOD activity, *CAT, SOD, GPx*, and *Nrf2* mRNA expression level (*p* < 0.05). The proportion of *Turicibacter* had a positive correlation with *IL-1β, IL-6, TLR4*, and *Keap1* mRNA expression level (*p* < 0.05), and had a negative correlation with TAC, CAT, and SOD activity, GSH content, *CAT, SOD, GPx*, and *Nrf2* mRNA expression level (*p* < 0.05). The proportion of *Subdoligranulum* had a positive correlation with *IL-1β* and *Keap1* mRNA expression level (*p* < 0.05), and had a negative correlation with CAT and SOD activity, *CAT, GPx*, and *Nrf2* mRNA expression level (*p* < 0.05). The proportion of *Ruminococcus_torques_group* had a positive correlation with TNF-α content, *IL-1β, IL-6*, and *Keap1* mRNA expression level (*p* < 0.05), and had a negative correlation with TAC and CAT activity, *CAT, GPx*, and *Nrf2* mRNA expression level (*p* < 0.05). The proportion of *Lactococcus* had a positive correlation with *NF-κB p65* mRNA expression level (*p* < 0.05). The proportion of *Veillonella* had a positive correlation with IL-6 content and *NF-κB p65* mRNA expression level (*p* < 0.05). The proportion of *Enterococcus* had a positive correlation with TNF-α content, and had a negative correlation with *GPx* mRNA expression level (*p* < 0.05). The proportion of *Rothia* had a negative correlation with *GPx* mRNA expression level (*p* < 0.01). The proportion of *Macrococcus* had a positive correlation with *IL-6* mRNA expression level (*p* < 0.05), and had a negative correlation with CAT activity, *GPx* and *Nrf2* mRNA expression level (*p* < 0.05). The proportion of *Corynebacterium* had a negative correlation with *Nrf2* mRNA expression level (*p* < 0.05). The proportion of *Candidatus_Arthromitus* had a negative correlation with TAC ability, *NF-κB p65* mRNA expression level (*p* < 0.05).

## 4 Discussion

Previous studies found that *Artemisia* plant polysaccharides (such as *Artemisia argyi* and *Artemisia ordosica*) improved growth performance of broilers (Zhang et al., 2022b; Du et al., 2023). In the present study, dietary supplementation with AAP significantly increased BW on d 42, ADFI and ADG on d 36–42 in broilers. This is similar to our previous study, which found that dietary supplementation with *Artemisia annua* L. water extract increased the final body weight and feed efficiency of broilers (Guo et al., 2023). Furthermore, we found that oral administration of *E. coli* significantly reduced BW, ADG, and ADFI, and increased FCR of broilers in the present study. This is consistent with the study of Wu et al. (2021), who found that *E. coli*-challenge decreased ADG, ADFI, and BW in broilers. It is noteworthy that, in the present experiment, BW, ADG, ADFI and FCR of broilers in AAP + *E. coli* group were not different from those in control group, suggesting that dietary supplementation with AAP alleviated the decline in growth performance caused by *E. coli*-challenge. Moreover, Jahanian et al. (2021) observed that *E. coli* O78:K80-infected broilers significantly decreased ADFI and ADG during the trial period, however, dietary addition of silymarin significantly improved ADG in finisher and throughout the trial period. In the current study, dietary inclusion AAP increased growth performance of broilers, which might be due to its stimulant effect on appetite, improvement action on nutrient digestibility, and the consequent decrease in the gastrointestinal retention of food in birds. Simultaneously, we found that the apparent metabolic rate of EE (d 42), Ca (d 42) and P (d 21) in AAP + *E. coli* group was significantly higher than that in *E. coli* group. The nutrient metabolic rate is an important indicator for evaluating the digestion and absorption of nutrients in poultry. Its level is directly impacted by the growth performance of poultry and reflected the diet’s nutritional value. Previous experiment showed that *Astragalus* polysaccharides and *Glycyrrhiza* polysaccharides increased diets’ apparent metabolic rate in broilers (Qiao et al., 2022a). Previous studies have shown that *Radix rehmanniae preparata* polysaccharide supplementation can enhance mineral absorption in broilers (Yang et al., 2023), thus interact with Ca and P to promote the absorption of both. Therefore, we preliminarily speculated that dietary AAP could improve the growth performance of broilers by increasing the nutrient apparent metabolic rate.

Intestinal permeability is one of the indicators that indirectly reflect the damage of the intestinal mucosal barrier. Among them, the representative indicators include DAO, D-LA and ET (Guo et al., 2022b). In the present study, we observed that the activity of DAO and ET (d 42) in serum of broilers was significantly higher than that in the control group after oral administration of *E. coli*, indicating *E. coli* caused intestinal damage in broilers. As reported by Wu et al. (2021), *E. coli*-challenged broilers significantly increased the serum concentration of DAO and LPS compared with the unchallenged birds over the whole infection period (d 14 and 21). However, in our study, there was no difference in DAO activity between AAP + *E. coli* group and the control group, indicating that dietary supplementation AAP alleviated the damage caused by *E. coli*-challenge. In addition, intestinal permeability is closely related to intestinal villi morphology. When the intestine is damaged, the intestinal villi shrink or fall off, and the permeability of the intestine increases (Song et al., 2023; Zhang et al., 2023). In the present study, we found that *E. coli*-challenged broilers significantly reduced the VH and VH/CD of jejunum villi, but there was no difference between the AAP + *E. coli* group and the control group, indicating that dietary inclusion of AAP alleviated the damage of intestinal villi morphology caused by *E. coli*-challenge. This result is consistent with the finding reported by Liu et al. (2018), who found that dietary supplementation *Achyranthes bidentata* polysaccharide could significantly increase the VH and VH/CD of broilers caused by *E. coli* K88-challenge, indicating that polysaccharides could improve the intestine villus morphology of the birds. Tight junction protein is an important indicator of intestinal permeability. In the current study, we found that oral administration of *E. coli* significantly reduced mRNA expression level of *Claudin-1* and *Occludin* in broilers, which was consistent with the mentioned increase in the level of DAO and D-LA, suggesting that *E. coli*-challenge leads to increase in intestinal permeability and damage of tight junction proteins, ultimately resulting in impairment of intestinal mucosal integrity. Similar to the findings reported by Wu et al. (2022b), who found that the ileum mRNA level of *Occludin*, *Claudin* and *ZO-1* were significantly decreased in broilers by *E. coli*-challenge, however, dietary hydrolyzed wheat gluten supplementation ameliorated the decrease of tight junction protein mRNA expression caused by *E. coli*-challenge. Besides, it was reported that dietary supplementation of *Astragalus membranaceus* polysaccharides and *Glycyrrhiza uralensis* polysaccharides significantly increased the intestinal *ZO-1*, *Claudin-1* and *Occludin* mRNA expression level of broilers (Qiao et al., 2022b). It is noteworthy that the AAP + *E. coli* group showed no difference in tight junction protein mRNA level compared with the control group in the current study. Similar outcomes were observed by Liu et al. (2023) that dietary addition of *Enteromorpha prolifera* polysaccharides upregulated the mRNA expression level of *Occludin*, *ZO-1* in jejunum of broilers under heat stress. Thus, dietary supplementation AAP mitigated the decrease in tight junction protein mRNA expression level caused by *E. coli*-challenge. Mucin-2 is an important component of the intestinal mucous layer and plays an important role in resistance to pathogen invasion. It was previously reported that dietary supplementation of plant polysaccharides significantly increased the mRNA level of intestinal *Mucin-2* in broilers (Qiao et al., 2022b). In the present study, we found that dietary supplementation with AAP effectively mitigated the decrease of *Mucin-2* mRNA expression level in the intestine of broilers caused by *E. coli*-challenge. Moreover, it has been reported that prebiotics can alleviate the decrease of *Mucin-2* mRNA expression level in broilers caused by *E. coli*-challenge (Huang et al., 2019).

The immune function of the body is essential for maintaining the health of the body. In this study, we observed that dietary inclusion AAP significantly increased the serum IgA content of broilers. Immunoglobulins are an important part of the body’s immune system and play an important role in resisting pathogen invasion. Both IgG and IgA are secreted by B lymphocytes as a part of humoral immunity. The present study showed that dietary supplementation AAP alleviated the decrease of serum IgM and jejunum IgG content in broilers caused by *E. coli*-challenge. In addition, Zheng et al. (2023) found that dietary Yupingfeng polysaccharides increased serum IgA, IgM and IgG level. It is well known that polysaccharides can enhance the secretion of B lymphocytes (Wu et al., 2022a), which is consistent with the results of the present investigation. Besides, it was reported that dietary addition of *Achyranthes bidentata* polysaccharides alleviated the decrease of sIgA content in intestinal mucosa caused by *E. coli* K88-challenge (Liu et al., 2018). Furthermore, in our study, we found that *E. coli*-challenged broilers significantly increased serum IL-6 and jejunum IL-1β and IL-6 content on d 42, however, dietary supplementation with AAP could effectively alleviate the increase of the proinflammatory cytokines caused *E. coli*-challenge. Furthermore, inflammatory factors like IL and TNF activate cell membrane receptors, initiating signal cascades that activate transcription factors, leading to increased gene transcription. They also affect mRNA-binding proteins, altering mRNA stability and degradation, influencing mRNA expression. Additionally, they modulate translation regulators, impacting mRNA translation efficiency and protein synthesis rate (Stumpo et al., 2010). In the current study, oral administration of *E. coli* significantly increased the mRNA level of *IL-1β*, *IL-6* and *TNF-α* in jejunum, and dietary supplementation of AAP alleviated the up-regulation of mRNA level, and the trend was consistent with the content of inflammatory factors. Previous research found that dietary *Astragalus* polysaccharide significantly decreased the mRNA level of *TNF-α* and *IL-1β* in chickens challenged by *E. coli* (Su et al., 2019). Hence, dietary supplementation AAP alleviated the upregulation of inflammatory factors’ mRNA caused by *E. coli*-challenge. Presumably, the upregulation of pro-inflammatory factors may be regulated through signaling pathways such as TLR4 and NF-κB. In the current study, oral administration of *E. coli* significantly increased the mRNA level of *TLR4* and *MyD88* in jejunum, and dietary supplementation of AAP alleviated the upregulation of mRNA expression level. These results are a line with those found by Wu et al. (2022b), who reported that dietary hydrolyzed wheat gluten supplementation down-regulated the elevation of ileal *TLR4* mRNA level caused by *E. coli* O78-challenge in broilers. It might be related to the anti-inflammatory activity of arabinose, galactose and fucoidan in plant-derived polysaccharides (Wang et al., 2023). In addition, *in vitro* experiment found the *E. coli*-induced intestinal barrier dysfunction was alleviated by seaweed polysaccharide supplementation via the inhibition of the NF-κB pathway (*TLR4*, *MyD88*, *IκBα* and *p65* mRNA level) and inflammatory cytokines (IL-6 and TNF-α) production of IPEC-J2 cells (Guo et al., 2021). Another *in vitro* experiment showed that AAP significantly inhibited the production of IL-6 and TNF-α in murine RAW 264.7 macrophages stimulated by lipopolysaccharide (LPS) (Zhang et al., 2022a). Remarkably, our previous study found that *Artemisia ordosica* polysaccharides decreased LPS-induced over-production of IL-1β and IL-6 through suppressing TLR4/NF-κB pathway, and alleviated LPS-induced decreasing of TAC, CAT and GPx activity by activating Nrf2/Keap1 pathway, which ultimately improved jejunum morphology (Xing et al., 2023). LPS on the cell wall of *E. coli* is one of the main pathogenic components, and can be specifically recognized by TLR4 on the surface of the cell membrane, induce intestinal epithelial cells to release a large number of inflammatory factors, and stimulate the body to produce excessive ROS, thus generating oxidative stress.

Our study found that oral administration of *E coli* decreased the activity of serum GPx, jejunum TAC, CAT and SOD, and increased the content of MDA in jejunum, suggesting that *E. coli*-challenge destroyed the intestinal antioxidant system and caused oxidative stress to a certain extent. Also, dietary inclusion of AAP alleviated the decrease of antioxidant enzyme activity and the increase of MDA content caused by *E. coli*-challenge in the current study. These results resonate with those reported by Dong et al. (2019), who observed that dietary addition of *Camellia oleifera* seed extract alleviated the decrease of serum GPx and SOD activity and the increase of MDA content in broilers caused by *E. coli* K88-challenge. Moreover, dietary supplementation of AAP alleviated the decrease of mRNA expression level of *CAT*, *SOD*, *GPx* and *Nrf2* and the increase of mRNA level of *Keap1* in jejunum caused by *E. coli*-challenge in our study. This is consistent with the change of antioxidant enzyme activity. Intriguingly, our previous study found that *A. annua* aqueous extract promoted the intestinal immune and antioxidant function of broilers (Guo et al., 2022a). Based on the above-mentioned results, we preliminarily speculated that AAP might alleviate intestinal oxidative damage caused by *E. coli*-challenge through Nrf2 pathway. Moreover, it might also be associated to the *in vitro* antioxidant activity of AAP, which has been reported to have significant OH^•^, DPPH^•^ and ABTS^•+^ free radical scavenging capacity (Zhang et al., 2022a). Therefore, dietary supplementation with AAP could alleviate intestinal oxidative damage caused by *E. coli*-challenge.

Intestinal microbiota is closely related to various physiological functions such as growth performance, metabolism and immunity in poultry (Díaz Carrasco et al., 2022). And we previously summarized that plant-derived polysaccharides could regulate intestinal health by improving intestinal microbial barrier (Guo et al., 2022b). So far, it has not been reported that *A. annua* polysaccharide regulates intestinal microflora. In the current study, 16S rRNA sequencing technology was used to analyze the jejunal microbiota of broilers to explore the effect of AAP on the taxonomic composition of the gut microbial community under *E. coli* challenge. We found that the jejunum microbial α diversity Simpson and Ace index in *E. coli* group had a trend of increasing and decreasing on d 21, respectively. The higher the Simpson index value, the lower the community diversity. The greater the Ace index, the richer the community species. Therefore, it indicated that *E. coli*-challenge decreased jejunum α diversity. However, on d 42 of the trial, different results were presented. Throughout the growth process of broiler chickens, significant changes are undergone by their intestinal structure, function, and digestive capacity with age. The two time points of d 21 and d 42 represented different growth stages, which are affected by the diversity of microorganisms. The increase in nutritional demand with age is affected by the nutritional sources and growth and reproduction of intestinal microorganisms. Difference between the earlier and later stages of intestinal development are led to significant difference in the α diversity of the jejunal microbial community. Mao et al. (2022) found that the α diversity of the ileal microbiota of broilers in d 21 and d 42 was different. In our study, the Sobs, Chao, Shannon and Ace index of broiler jejunum microbiota were increased significantly by *E. coli*-challenged, while Simpson and Coverage index were decreased on d 42. Contrary to our study, Pham et al. (2023) found that *E. coli* O78-challenge reduced the Chao and Ace index. Interestingly, there was no difference in the aforementioned α diversity index between the AAP + *E. coli* group and the control group. The results indicated that dietary AAP supplementation could improve α diversity of jejunum microbe in broilers challenged by *E. coli*.

The results of β diversity showed that the microbiota structure was significantly different among groups. The taxonomical composition analysis showed that *Firmicutes* were the most dominant phylum in jejunum of broilers, accounting for 89.82, 91.08, 95.13, and 92.92% on d 21, 98.24, 98.29, 96.17, and 95.06% on d 42 in groups control, AAP, *E. coli*, AAP + *E. coli*, respectively. However, the results demonstrated that no significant difference was observed among the four groups. In the current study, at the phylum level, we found a significant increase in the abundance of jejunum *Desulfobacterota* in the AAP group and the AAP + *E. coli* group on d 21. It is reported that *Desulfobacterota* is correlated with the level of inflammatory factors (Zhang et al., 2023). Different from previous period, at the phylum level, we found that the abundance of *Bacteroidota* in the jejunum contents of the AAP + *E. coli* group was significantly higher than that of other groups on d 42. *Bacteroides* is considered to be a beneficial bacterium, and its increased abundance might be due to a slowing down of intestinal absorption of AAP caused by *E. coli*-challenge, which promotes the growth of Bacteroides. The taxonomical composition analysis showed that *Lactobacillus* were the most dominant genus in the jejunum of broilers, accounting for 70.34, 70.38, 80.91, and 81.67% on d 21, 88.77, 88.13, 55.00, and 61.27% on d 42 in groups control, AAP, *E. coli*, AAP + *E. coli*, respectively. *E. coli*-challenge might stimulate the growth of Lactobacillus, but the difference is not significant on d 21. Interestingly, the results were different on d 42. The two time points of d 21 and d 42 represented different growth stages, which are likely accompanied by adjustments in the structure and function of the digestive system, thus influencing the microbial flora. In our study, the abundance of *Lactobacillus* decreased significantly under the condition of *E. coli*-challenge on d 42. Previously, Zeng et al. (2023) reported that *E. coli* K88-challenge reduced the number of *Lactobacillus* in jejunum, ileum and colon of piglets. Previous studies showed that Yupingfeng polysaccharides increased the abundance of *Lactobacillus* genus in duodenum of broilers (Zheng et al., 2023). Coincidentally, LEfSe analysis showed that addition of AAP increased the number of dominant bacteria *Lactobacillales* in the jejunum at the order level in our study. Furthermore, it was found that the proportion of *Lactobacillus* was negatively correlated with the level of inflammatory factors, but positively correlated with the level of antioxidants, indicating that the proliferation of *Lactobacillus* could be promoted by AAP and the inflammatory response could be reduced. Besides, at the genus level, we found that the abundance of *Megasphaera* and *Megamonas* in jejunum contents of AAP group and AAP + *E. coli* group was significantly reduced. *Megasphaera* and *Megamonas* can ferment polysaccharides to produce SCFAs, such as acetic acid, propionic acid and butyric acid, further inhibiting the growth of pathogenic microorganisms (Liu et al., 2023). In the current study, the abundance of *Megasphaera* and *Megamonas* was decreased, which might be partially consumed during the fermentation of polysaccharides. It has previously been reported that dietary supplementation with *Antrodia cinnamomea* polysaccharide mitigated the decrease in the abundance of *Megamonas* and *Ruminococcus_torques_group* in cecal microbiota of broilers challenged by LPS (Ye et al., 2022), similar to the findings in our experiment. *Parabacteroides* regulate intestinal immune response and are positively correlated with IgG (Qiao et al., 2022b). In the present study, the abundance of *Parabacteroides* in jejunum of broilers was significantly reduced under the condition of *E. coli* challenge, indicating that *E. coli* challenge disrupted the microbiological balance. *Ralstonia* is a gram-negative bacterium (Deng et al., 2021), and dietary supplementation with AAP significantly reduced its abundance in our study. Pham et al. (2023) also found that *E. coli* O78-challenge increased the abundance of cecum *Oscillospira* in broilers, which is similar to the results of this experiment. However, Qiao et al. (2022a) found that *Oscillospira* was positively correlated with *GPx* mRNA expression level, and dietary supplementation of polysaccharides derived from *astragalus* and *glycyrrhiza* increased *Oscillospira* abundance in conventional feeding. We found that *Faecalibacterium* and *Negativibacillus* are mainly enriched in *E. coli* group (Figure 12). Previous experiment found that dietary supplementation of polysaccharides derived from *astragalus* and *glycyrrhiza* decreased the relative abundance of *Faecalibacterium*, and was positively correlated with MDA, but negatively correlated with T-AOC, GPx, SOD1 mRNA expression, indicating that the polysaccharides could improve antioxidant function by modulating gut microbiota in broilers (Qiao et al., 2022a). Previous studies showed that dietary supplementation of *astragalus* polysaccharide decreased the abundance of *Negativibacillus* in cecum of broilers, thereby improving intestinal health (Wang et al., 2022). Similarly, dietary AAP reduced the abundance of *Faecalibacterium* and *Negativibacillus* in jejunum of broilers challenged by *E. coli* in our study. *UCG-005* is closely related to inflammation (Song et al., 2023). In our experiment, we observed a positive correlation between the mRNA expression levels of *IL-1β*, *IL-6*, *TLR4*, and *MyD88* and the proportion of *UCG-005*. Furthermore, *UCG-005* was found to be enriched in the *E. coli* group, as depicted in Figures 12, 13D. Dietary AAP reduced the abundance of *UCG-005* under *E. coli*-challenged, suggesting that AAP might reduce the expression of intestinal pro-inflammatory factors by inhibiting the proliferation of *UCG-005*. *Blautia* is related to the immune response of the gut, and is also involved in regulating the body’s physiological responses (Li et al., 2022). Compared with the *E. coli* group, the abundance of *Blautia* in the AAP + *E. coli* group was down-regulated, indicating that AAP plays a role in the regulation of intestinal homeostasis. Song et al. (2022) found that dietary supplementation of *astragalus* polysaccharide alleviated necrotic enteritis-induced intestinal inflammatory damage by increasing the abundance of *Romboutsia* in the ileum. In our experiment, we observed a positive correlation between the levels of *IL-1β*, *IL-6*, *TLR4*, and *MyD88* and the proportion of *Romboutsia*. Additionally, it was noted that *Romboutsia* exhibited no enrichment in the AAP group (Figure 13D), suggesting a potential inhibitory effect of AAP on its proliferation.

In this study, we found that the abundance of various beneficial bacteria in the *E. coli* group was up-regulated, such as *Blautia*, *Megamonas* and *Christensenellaceae R-7 group*, which may inhibit the reproduction of *E. coli* by participating in some different metabolic pathways, such as TCA cycle, butyrate metabolism, propionic acid metabolism (Li et al., 2022). Furthermore, the expansion of numerous beneficial bacteria indicated that *E. coli* resulted in a microbial imbalance in the gut, and prompting the production of a large number of beneficial bacteria in the intestine to regain the balance of microbiota and inhibit the growth of pathogenic bacteria (Li et al., 2021). However, the abundance of these beneficial bacteria in the AAP + *E. coli* group was reduced, which might be attributed to the pre-protective effect of AAP to restore intestinal microbiota balance as soon as possible. Moreover, it might also regulate the balance of microecology through other ways. Our previous studies have shown that *Artemisia ordosica* polysaccharide can prevent intestinal inflammation in broilers by inhibiting TLR4/NF-κB and activating Nrf2/Keap1 pathway (Xing et al., 2023). Furthermore, plant polysaccharides can also promote intestinal peristalsis and defecation, help to expel harmful substances and metabolites in poultry, further reduce the intestinal burden, and is conducive to the growth and reproduction of beneficial bacteria (Sundar et al., 2020). In addition, it was reported that whether polysaccharides can play an antibacterial role is related to the molecular weight of polysaccharides, and large molecular weight polysaccharides can inhibit harmful bacteria such as *E. coli* (Wang et al., 2023). It might explain the relatively low abundance of bacteria detected in the AAP + *E. coli* group. We found that the microbiota changes of d 21 and d 42 were different (Figure 13), which might be related to the age and feed intake of broilers at different periods (Konieczka et al., 2017). In general, *E. coli*-challenge destroyed the balance of jejunum microbiota in broilers, and dietary addition of AAP could restore the balance of microbe.

## 5 Conclusion

In summary, based on growth performance, apparent nutrient metabolic rate, intestinal permeability, immune response, intestinal morphology, antioxidant function, jejunal microbiota, we conclude that *E. coli*-challenge impaired intestinal health, and eventually decreased the growth performance of broilers. Dietary AAP supplementation can effectively reduce intestinal damage, improve the intestinal barrier function, enhance immune and antioxidant function, re-shape intestinal microbiota. These results suggest that AAP may be an effective option for the prevention of *E. coli* in poultry breeding.

## Data availability statement

The datasets presented in this study can be found in the NCBI repository, accession number PRJNA1098622.

## Ethics statement

The animal study was approved by Inner Mongolia Agricultural University Animal Care and Use Committee, Hohhot, P. R. China (approval number: NND2021090). The study was conducted in accordance with the local legislation and institutional requirements.

## Author contributions

SG: Conceptualization, Formal analysis, Methodology, Visualization, Writing – original draft. BS: Supervision, Validation, Writing – review & editing. YyX: Writing – review & editing. YqX: Resources, Validation, Writing – review & editing. XJ: Data curation, Validation, Writing – original draft. LH: Supervision, Writing – original draft. SZ: Investigation, Writing – review & editing. MQ: Writing – review & editing. SY: Validation, Writing – original draft.

## Glossary

AAP: *Artemisia annua* L. polysaccharide
ADFI: Average daily feed intake
ADG: Average daily gain
BW: Body weight
Ca: Calcium
CAT: Catalase
CD: Crypt depth
CON: Control
CP: Crude protein
DAO: Diamine oxidase
D-LA: D-lactate
DM: Dry matter
EE: Ether extract
*E. coli*: *Escherichia. coli*
ELISA: Enzyme-linked immunosorbent assay
ET: Endotoxin
FCR: Feed conversion ratio
GPx: Glutathione peroxidase
GSH: Glutathione
Ig: Immunoglobulin
IL: Interleukin
JAM-2: Junctional adhesion molecule-2
Keap1: Kelch like ECH associated protein 1
MDA: Malondialdehyde
MyD88: Myeloid differentiation primary response 88
NF-κB: Nuclear factor-κB
Nrf2: Nuclear factor erythroid-2-related factor 2
P: Phosphorus
RT-PCR: Reverse transcription-polymerase chain reaction
sIgA: Secretory immunoglobulin A
SOD: Superoxide dismutase
TAC: Total antioxidant capacity
TNF-α: Tumor necrosis factor α
TLR: Toll-like receptor
VH: Villus height
VH/CD: Villus height/crypt depth
ZO-1: Zonula occludens-1

## References

[ref1] Berkes J. Viswanathan V. K. Savkovic S. D. Hecht G. (2003). Intestinal epithelial responses to enteric pathogens: effects on the tight junction barrier, ion transport, and inflammation. Gut 52, 439–451. doi: 10.1136/gut.52.3.439, PMID: 12584232 PMC1773546

[ref2] Chebbac K. Benziane Ouaritini Z. El Moussaoui A. Chalkha M. Lafraxo S. Bin Jardan Y. A.. (2023). Antimicrobial and antioxidant properties of chemically analyzed essential oil of *Artemisia annua* L. (Asteraceae) native to Mediterranean area. Life 13:807. doi: 10.3390/life13030807, PMID: 36983962 PMC10055474

[ref3] Chinese Ministry of Agriculture. Feeding standard of chicken, China (NY/T 33-2004). (2004). Hunan Feed. Liu G Xiong B Su J Ji C Diao Q Liu H. (Eds.) Beijing: Ministry of Agriculture of the People’s Republic of China 4, 19–27.

[ref4] Choi E. Y. Choi J. O. Park C. Y. Kim S. H. Kim D. (2020). Water extract of *Artemisia annua* L. exhibits Hepatoprotective effects through improvement of lipid accumulation and oxidative stress-induced cytotoxicity. J. Med. Food 23, 1312–1322. doi: 10.1089/jmf.2020.4696, PMID: 33202166

[ref5] Croxen M. A. Law R. J. Scholz R. Keeney K. M. Wlodarska M. Finlay B. B. (2013). Recent advances in understanding enteric pathogenic *Escherichia coli*. Clin. Microbiol. Rev. 26, 822–880. doi: 10.1128/CMR.00022-13, PMID: 24092857 PMC3811233

[ref6] Cui Y. Sun W. Li Q. Wang K. Wang Y. Lv F.. (2022). Effects of *caulis Spatholobi* polysaccharide on immunity, intestinal mucosal barrier function, and intestinal microbiota in cyclophosphamide-induced immunosuppressive chickens. Front. Vet. Sci. 9:833842. doi: 10.3389/fvets.2022.833842, PMID: 35372558 PMC8972122

[ref7] Das S. Vörös-Horváth B. Bencsik T. Micalizzi G. Mondello L. Horváth G.. (2020). Antimicrobial activity of different *Artemisia* essential oil formulations. Molecules 25:2390. doi: 10.3390/MOLECULES25102390, PMID: 32455592 PMC7287661

[ref8] Deng S. Xu Q. Fu Y. Liang L. Wu Y. Peng F.. (2021). Genomic analysis of a novel phage infecting the Turkey pathogen *Escherichia coli* APEC O78 and its Endolysin activity. Viruses 13:1034. doi: 10.3390/v13061034, PMID: 34072620 PMC8229158

[ref9] Díaz Carrasco J. M. Redondo L. M. Casanova N. A. Fernández Miyakawa M. E., (2022). The role of farm environment and Management in Shaping the gut microbiota of poultry. Gut microbiota, immunity, and health in production animals. Cham: Springer International Publishing. 193–224

[ref10] Ding F. Ma T. Hao M. Wang Q. Chen S. Wang D.. (2020). Mapping worldwide environmental suitability for *Artemisia annua* L. Sustain. For. 12:1309. doi: 10.3390/su12041309

[ref11] Dong Z. L. Wang Y. W. Song D. Wang W. W. Liu K. B. Wang L.. (2019). Effects of microencapsulated probiotics and plant extract on antioxidant ability, immune status and caecal microflora in *Escherichia coli* K88-challenged broiler chickens. Food Agr. Immunol. 30, 1123–1134. doi: 10.1080/09540105.2019.1664419

[ref12] Du H. Xing Y. Jin X. Yan S. Shi B. (2023). Effects of *Artemisia ordosica* polysaccharide on growth performance and antioxidant capacity in broilers. J. Appl. Anim. Res. 51, 92–101. doi: 10.1080/09712119.2022.2158093

[ref13] Fu C. Yu P. Wang M. Qiu F. (2020). Phytochemical analysis and geographic assessment of flavonoids, coumarins and sesquiterpenes in *Artemisia annua* L. based on HPLC-DAD quantification and LC-ESI-QTOF-MS/MS confirmation. Food Chem. 312:126070. doi: 10.1016/j.foodchem.2019.126070, PMID: 31911352

[ref14] Gomes T. A. Elias W. P. Scaletsky I. C. Guth B. E. Rodrigues J. F. Piazza R. M.. (2016). Diarrheagenic *Escherichia coli*. Braz. J. Microbiol. 47, 3–30. doi: 10.1016/j.bjm.2016.10.015, PMID: 27866935 PMC5156508

[ref15] Graham I. A. Czechowski T. Rinaldi M. A. Famodimu M. T. Van Veelen M. Larson T. R.. (2019). Flavonoid versus artemisinin anti-malarial activity in *Artemisia annua* whole-leaf extracts. Front. Plant Sci. 10:984. doi: 10.3389/fpls.2019.00984, PMID: 31417596 PMC6683762

[ref16] Guo X. Chen J. Yang J. He Q. Luo B. Lu Y.. (2021). Seaweed polysaccharide mitigates intestinal barrier dysfunction induced by enterotoxigenic *Escherichia coli* through NF-κB pathway suppression in porcine intestinal epithelial cells. J. Anim. Physiol. Anim. Nutr. 105, 1063–1074. doi: 10.1111/jpn.13540, PMID: 33817860

[ref17] Guo S. Ma J. Xing Y. Shi L. Zhang L. Xu Y.. (2022a). *Artemisia annua* L. aqueous extract promotes intestine immunity and antioxidant function in broilers. Front. Vet. Sci. 9:934021. doi: 10.3389/fvets.2022.934021, PMID: 35873687 PMC9304935

[ref18] Guo S. Ma J. Xing Y. Xu Y. Jin X. Yan S.. (2023). Effects of *Artemisia annua* L. water extract on growth performance and intestinal related indicators in broilers. J. Poult. Sci. 60:2023024. doi: 10.2141/jpsa.2023024, PMID: 37711228 PMC10495255

[ref19] Guo S. Xing Y. Xu Y. Jin X. Yan S. Shi B. (2022b). Progress of studies on plant-derived polysaccharides affecting intestinal barrier function in poultry. Animals 12:3205. doi: 10.3390/ani12223205, PMID: 36428432 PMC9686483

[ref20] Han X. Chai Y. Lv C. Chen Q. Liu J. Wang Y.. (2022). Sesquiterpenes from *Artemisia annua* and their cytotoxic activities. Molecules 27:5079. doi: 10.3390/molecules27165079, PMID: 36014318 PMC9414659

[ref21] Huang L. Luo L. Zhang Y. Wang Z. Xia Z. (2019). Effects of the dietary probiotic, *Enterococcus faecium* NCIMB11181, on the intestinal barrier and system immune status in *Escherichia coli* O78-challenged broiler chickens. Probiotics Antimicrob. Proteins 11, 946–956. doi: 10.1007/s12602-018-9434-7, PMID: 29948799 PMC6695369

[ref22] Jahanian E. Mahdavi A. H. Jahanian R. (2021). Silymarin improved the growth performance via modulating the microbiota and mucosal immunity in *Escherichia coli*-challenged broiler chicks. Livest. Sci. 249:104529. doi: 10.1016/j.livsci.2021.104529

[ref23] Konieczka P. Barszcz M. Choct M. Smulikowska S. (2017). The interactive effect of dietary n-6: n-3 fatty acid ratio and vitamin E level on tissue lipid peroxidation, DNA damage in intestinal epithelial cells, and gut morphology in chickens of different ages. Poult. Sci. 97, 149–158. doi: 10.3382/ps/pex274, PMID: 29077918 PMC5850597

[ref24] Kumari M. Gupta R. P. Bagri P. Singh R. (2023). Immunopathological studies on *Escherichia coli* infected broiler chickens fed on *Aloe vera* leaf extract. Vet. Immunol. Immunopathol. 258:110562. doi: 10.1016/j.vetimm.2023.110562, PMID: 36801725

[ref25] Li S. Lin R. Chen J. Hussain R. Zhang S. Su Y.. (2022). Integrated gut microbiota and metabolomic analysis reveals immunomodulatory effects of Echinacea extract and *Astragalus* polysaccharides. Front. Vet. Sci. 9:971058. doi: 10.3389/fvets.2022.971058, PMID: 36118329 PMC9478787

[ref26] Li Y. Xia S. Jiang X. Feng C. Gong S. Ma J.. (2021). Gut microbiota and diarrhea: An updated review. Front. Cell. Infect. Microbiol. 11:625210. doi: 10.3389/fcimb.2021.625210, PMID: 33937093 PMC8082445

[ref27] Liu W. Liu H. Wang Y. Zhao Z. Balasubramanian B. Jha R. (2023). Effects of *Enteromorpha prolifera* polysaccharides on growth performance, intestinal barrier function and cecal microbiota in yellow-feathered broilers under heat stress. J. Anim. Sci. Biotechnol. 14:132. doi: 10.1186/s40104-023-00932-2, PMID: 37814279 PMC10563363

[ref28] Liu Z. Wang X. Ou S. Arowolo M. A. Hou D. X. He J. (2018). Effects of *Achyranthes bidentata* polysaccharides on intestinal morphology, immune response, and gut microbiome in yellow broiler chickens challenged with *Escherichia coli* K88. Polymers 10:1233. doi: 10.3390/polym10111233, PMID: 30961158 PMC6401798

[ref29] Mao J. Wang Y. Wang W. Duan T. Yin N. Guo T.. (2022). Effects of *Taraxacum mongolicum hand.-Mazz*. (dandelion) on growth performance, expression of genes coding for tight junction protein and mucin, microbiota composition and short chain fatty acids in ileum of broiler chickens. BMC Vet. Res. 18:180. doi: 10.1186/s12917-022-03278-5, PMID: 35568942 PMC9107267

[ref30] Pham V. H. Abbas W. Huang J. Guo F. Zhang K. Kong L.. (2023). Dietary coated essential oil and organic acid mixture supplementation improves health of broilers infected with avian pathogenic *Escherichia coli*. Anim. Nutr. 12, 245–262. doi: 10.1016/j.aninu.2022.09.010, PMID: 36712401 PMC9868345

[ref31] Qiao Y. Guo Y. Zhang W. Guo W. Oleksandr K. Bozhko N.. (2022a). Effects of compound polysaccharides derived from *Astragalus* and *Glycyrrhiza* on growth performance, meat quality and antioxidant function of broilers based on serum metabolomics and Cecal microbiota. Antioxidants 11:1872. doi: 10.3390/antiox11101872, PMID: 36290595 PMC9598874

[ref32] Qiao Y. Liu C. Guo Y. Zhang W. Guo W. Oleksandr K.. (2022b). Polysaccharides derived from *Astragalus membranaceus* and *Glycyrrhiza uralensis* improve growth performance of broilers by enhancing intestinal health and modulating gut microbiota. Poult. Sci. 101:101905. doi: 10.1016/j.psj.2022.101905, PMID: 35576745 PMC9117935

[ref33] Soares M. P. Cardoso I. L. Araújo F. E. De Angelis C. F. Mendes R. Mendes L. W.. (2022). Influences of the alcoholic extract of *Artemisia annua* on gastrointestinal microbiota and performance of Nile tilapia. Aquaculture 560:738521. doi: 10.1016/j.aquaculture.2022.738521

[ref34] Song Z. Cheng K. Zhang L. Wang T. (2017). Dietary supplementation of enzymatically treated *Artemisia annua* could alleviate the intestinal inflammatory response in heat-stressed broilers. J. Therm. Biol. 69, 184–190. doi: 10.1016/j.jtherbio.2017.07.015, PMID: 29037381

[ref35] Song Z. H. Cheng K. Zheng X. C. Ahmad H. Zhang L. L. Wang T. (2018). Effects of dietary supplementation with enzymatically treated *Artemisia annua* on growth performance, intestinal morphology, digestive enzyme activities, immunity, and antioxidant capacity of heat-stressed broilers. Poult. Sci. 97, 430–437. doi: 10.3382/ps/pex312 29077887

[ref36] Song B. He J. Pan X. Kong L. Xiao C. Keerqin C.. (2023). Dietary *Macleaya cordata* extract supplementation improves the growth performance and gut health of broiler chickens with necrotic enteritis. J. Anim. Sci. Biotechnol. 14:113. doi: 10.1186/s40104-023-00916-2, PMID: 37674220 PMC10483844

[ref37] Song B. Li P. Yan S. Liu Y. Gao M. Lv H.. (2022). Effects of dietary *Astragalus* polysaccharide supplementation on the Th17/Treg balance and the gut microbiota of broiler chickens challenged with necrotic enteritis. Front. Immunol. 13:781934. doi: 10.3389/fimmu.2022.781934, PMID: 35265068 PMC8899652

[ref38] Stan R. L. (2020). *Artemisia Annua* L. extract: a new Phytoproduct with sod-like and antitumour activity. Farmacia 68, 812–821. doi: 10.31925/farmacia.2020.5.6

[ref39] Stumpo D. J. Lai W. S. Blackshear P. J. (2010). Inflammation: cytokines and RNA-based regulation. Wiley Interdiscip. Rev. RNA. 1, 60–80. doi: 10.1002/wrna.1, PMID: 21956907 PMC3915420

[ref40] Su L. Wang J. Huang J. Zhao Y. Jiang H. Li H. (2019). Suppresses of *Astragalus* polysaccharide on *E. coli*-induced injured intestinal microvascular through TLR4-NF-κB signal pathways in chickens. Braz. J. Poultry Sci. 21, 1–8. doi: 10.1590/1806-9061-2018-0945

[ref41] Sundar V. Senthil Kumar K. A. Manickam V. Ramasamy T. (2020). Current trends in pharmacological approaches for treatment and management of acute pancreatitis—a review. J. Pharm. Pharmacol. 72, 761–775. doi: 10.1111/jphp.13229 32012276

[ref42] Wan X. Ahmad H. Zhang L. Wang Z. Wang T. (2018). Dietary enzymatically treated *Artemisia annua* L. improves meat quality, antioxidant capacity and energy status of breast muscle in heat-stressed broilers. J. Sci. Food Agric. 98, 3715–3721. doi: 10.1002/jsfa.8879 29315586

[ref43] Wan X. Zhang J. He J. Bai K. Zhang L. Wang T. (2017). Dietary enzymatically treated *Artemisia annua* L. supplementation alleviates liver oxidative injury of broilers reared under high ambient temperature. Int. J. Biometeorol. 61, 1629–1636. doi: 10.1007/s00484-017-1341-1, PMID: 28352954

[ref44] Wang W. Li Z. Han Q. Guo Y. Zhang B. D’Inca R. (2016). Dietary live yeast and mannan-oligosaccharide supplementation attenuate intestinal inflammation and barrier dysfunction induced by *Escherichia coli* in broilers. Br. J. Nutr. 116, 1878–1888. doi: 10.1017/S0007114516004116, PMID: 27989252

[ref45] Wang Q. Wang X. F. Xing T. Li J. L. Zhu X. D. Zhang L.. (2022). The combined impact of xylo-oligosaccharides and gamma-irradiated astragalus polysaccharides on the immune response, antioxidant capacity, and intestinal microbiota composition of broilers. Poult. Sci. 101:101996. doi: 10.1016/j.psj.2022.101996, PMID: 35841635 PMC9293642

[ref46] Wang Z. Zheng Y. Lai Z. Hu X. Wang L. Wang X.. (2023). Effect of monosaccharide composition and proportion on the bioactivity of polysaccharides: a review. Int. J. Biol. Macromol. 254:127955. doi: 10.1016/j.ijbiomac.2023.127955, PMID: 37944714

[ref47] Wu Q. G. Huang L. Y. Fan M. H. Chou G. X. Wang Y. L. (2023). Anti-inflammatory activities of monoterpene and Sesquiterpene glycosides from the aqueous extract of *Artemisia annua* L. Chem. Biodivers. 20:e202201237. doi: 10.1002/cbdv.202201237, PMID: 36740572

[ref48] Wu Y. Li N. Zhang T. Che Y. Duan K. Wang Y.. (2022a). Glycyrrhiza polysaccharides can improve and prolong the response of chickens to the Newcastle disease vaccine. Poult. Sci. 101:101549. doi: 10.1016/j.psj.2021.101549, PMID: 34837761 PMC8626840

[ref49] Wu Y. Wang W. Kim I. H. Yang Y. (2022b). Dietary hydrolyzed wheat gluten supplementation ameliorated intestinal barrier dysfunctions of broilers challenged with *Escherichia coli* O78. Poult. Sci. 101:101615. doi: 10.1016/j.psj.2021.101615, PMID: 34952261 PMC8715217

[ref50] Wu Y. Wu C. Che Y. Zhang T. Dai C. Nguyen A. D.. (2022c). Effects of Glycyrrhiza polysaccharides on Chickens' intestinal health and homeostasis. Front. Vet. Sci. 9:891429. doi: 10.3389/fvets.2022.891429, PMID: 35647094 PMC9134109

[ref51] Wu Z. Yang K. Zhang A. Chang W. Zheng A. Chen Z.. (2021). Effects of *Lactobacillus acidophilus* on the growth performance, immune response, and intestinal barrier function of broiler chickens challenged with *Escherichia coli* O157. Poult. Sci. 100:101323. doi: 10.1016/j.psj.2021.101323, PMID: 34280647 PMC8319008

[ref52] Xing Y. Y. Xu Y. Q. Jin X. Shi L. L. Guo S. W. Yan S. M.. (2020). Optimization extraction and characterization of *Artemisia ordosica* polysaccharide and its beneficial effects on antioxidant function and gut microbiota in rats. RSC Adv. 10, 26151–26164. doi: 10.1039/d0ra05063f, PMID: 35519751 PMC9055353

[ref53] Xing Y. Zheng Y. Yang S. Zhang L. Guo S. Shi L.. (2023). *Artemisia ordosica* polysaccharide ameliorated LPS-induced growth inhibition and intestinal injury in broilers through enhancing immune-regulation and antioxidant capacity. J. Nutr. Biochem. 115:109284. doi: 10.1016/j.jnutbio.2023.109284, PMID: 36828238

[ref54] Yan L. Xiong C. Xu P. Zhu J. Yang Z. Ren H.. (2019). Structural characterization and in vitro antitumor activity of a polysaccharide from *Artemisia annua* L. (Huang Huahao). Carbohydr. Polym. 213, 361–369. doi: 10.1016/j.carbpol.2019.02.081 30879680

[ref55] Yang B. Li X. Baran A. M. Abdel-Moneim A. E. (2023). Effects of dietary incorporation of Radix rehmanniae praeparata polysaccharide on growth performance, digestive physiology, blood metabolites, meat quality, and tibia characteristics in broiler chickens. Poult. Sci. 102:103150. doi: 10.1016/j.psj.2023.103150, PMID: 37871491 PMC10618489

[ref56] Ye J. Zhang C. Fan Q. Lin X. Wang Y. Azzam M.. (2022). *Antrodia cinnamomea* polysaccharide improves liver antioxidant, anti-inflammatory capacity, and cecal flora structure of slow-growing broiler breeds challenged with lipopolysaccharide. Front. Vet. Sci. 9:994782. doi: 10.3389/fvets.2022.994782, PMID: 36299632 PMC9588918

[ref57] Yegani M. Korver D. R. (2008). Factors affecting intestinal health in poultry. Poult. Sci. 87, 2052–2063. doi: 10.3382/ps.2008-00091, PMID: 18809868 PMC7107194

[ref58] Zeng Y. Li R. Dong Y. Yi D. Wu T. Wang L.. (2023). Dietary supplementation with Puerarin improves intestinal function in piglets challenged with *Escherichia coli* K88. Animals 13:1908. doi: 10.3390/ani13121908 37370417 PMC10295229

[ref59] Zhang S., (2023). Effects of *Artemisia annua* L. Polysaccharide on Immune and Antioxidant Functions of Broilers. Master’s Thesis. (In Chinese)

[ref60] Zhang L. Reddy N. Khoo C. S. Koyyalamudi S. R. (2022a). Structural characterization and in-vitro antioxidant and immunomodulatory activities of polysaccharide fractions isolated from *Artemisia annua* L. Molecules 27:3643. doi: 10.3390/molecules27113643, PMID: 35684579 PMC9182033

[ref61] Zhang J. Shu D. Cheng X. Tian T. Xiao K. Zhang D.. (2023). Effect of plant polysaccharides (Poria cocos and Astragalus polysaccharides) on immune responses and intestinal microbiota of Dabry's sturgeons. Biosci. Microb. Food H. 42, 243–253. doi: 10.12938/bmfh.2022-089, PMID: 37791344 PMC10542428

[ref62] Zhang L. Xing Y. Shi L. Guo S. Jin X. Xu Y.. (2022b). The effects of dietary supplementation of *Artemisia argyi* polysaccharide on immune and antioxidative functions in broilers. J. Appl. Anim. Res. 50, 587–597. doi: 10.1080/09712119.2022.2119982

[ref63] Zhang J. Yu H. Zhang H. Zhao Q. Si W. Qin Y.. (2023). Dietary Epimedium extract supplementation improves intestinal functions and alters gut microbiota in broilers. J. Anim. Sci. Biotechnol. 14:14. doi: 10.1186/s40104-022-00812-1, PMID: 36653873 PMC9847172

[ref64] Zhang S. Zhu C. Xie H. Wang L. Hu J. (2022c). Effect of Gan Cao (Glycyrrhiza uralensis Fisch) polysaccharide on growth performance, immune function, and gut microflora of broiler chickens. Poult. Sci. 101:102068. doi: 10.1016/j.psj.2022.102068, PMID: 36087472 PMC9465102

[ref65] Zheng W. Guan Y. Wu B. (2023). Effects of Yupingfeng polysaccharides as feed supplement on immune function and intestinal microbiome in chickens. Microorganisms 11:2774. doi: 10.3390/microorganisms11112774, PMID: 38004785 PMC10672924

